# ADP101 multifood oral immunotherapy for food-allergic patients: Harmony phase 1/2 randomized clinical trial

**DOI:** 10.1016/j.jacig.2024.100382

**Published:** 2024-12-10

**Authors:** Edwin H. Kim, Warner W. Carr, Amal H. Assa’ad, Shaila U. Gogate, Daniel H. Petroni, Thomas B. Casale, Mei-Lun Wang, Amy Sullivan, Amy M. Archer, Ouhong Wang, Cheri Piscia-Nichols, Lisa Tuomi, Olga Levin-Young, Ashley Dombkowski, Dana McClintock, Harmony investigators

**Affiliations:** a Department of Pediatrics, University of North Carolina School of Medicine, Chapel Hill, NC; b Allergy & Asthma Providers of Southern California, Mission Viejo, Calif; c Cincinnati Children’s Hospital Medical Center, Cincinnati, Ohio; d Colorado Allergy & Asthma Centers PC, Denver, Colo; e Seattle Allergy and Asthma Research Institute, Seattle, Wash; f University of South Florida, Tampa, Fla; g Alladapt Immunotherapeutics Inc, Menlo Park, Calif; h PointOH5 LLC, Boston, Mass

**Keywords:** Food allergy, multifood allergy, pediatric food allergy, allergy desensitization, oral immunotherapy, clinical trial

## Abstract

**Background:**

Oral immunotherapy is an established approach to desensitize the immune system in the context of allergic disease; however, the only currently approved product is for peanut allergy. ADP101 is a novel, pharmaceutical-grade, multifood oral immunotherapy in development to simultaneously treat single or multiple food allergies, containing allergenic proteins from 15 foods in equal parts by protein weight.

**Objective:**

The phase 1/2 Harmony trial (NCT04856865) evaluated efficacy and safety of ADP101 in participants with qualifying allergy to 1 to 5 foods in ADP101, defined as dose-limiting symptoms with a ≤100 mg challenge dose during double-blind, placebo-controlled food challenge (DBPCFC).

**Methods:**

Participants were randomized to low-dose (1500 mg/d; 100 mg protein per food) or high-dose (4500 mg/d; 300 mg protein per food) ADP101, or matched placebo, with dose escalation followed by daily maintenance dosing over 40 weeks. The primary endpoint was the proportion of participants tolerating a ≥600 mg challenge dose of a single qualifying food without dose-limiting symptoms at the Week 40 Exit DBPCFC (ie, responders).

**Results:**

In the primary analysis population (61 pediatric participants aged 4-17 years), a greater response rate was observed in both the high-dose ADP101 (55.0%) and low-dose ADP101 (38.1%) groups compared with pooled placebo (20.0%) (nominal *P* = .048, *P* = .306, respectively; adjusted for multiple comparisons, *P* = .097, *P* = .306, respectively). Desensitization to ≥2 foods was observed in individuals with multiple food allergies, as was desensitization at levels over 600 mg. ADP101-treated participants showed an overall reduction in skin-prick test reactivity, with an increase in maximum tolerated dose across the majority of foods tested. Adverse events were mostly mild or moderate, with no life-threatening events or deaths.

**Conclusions:**

The study did not meet its primary endpoint, but ADP101 demonstrated a favorable safety profile and increased the reactive threshold in DBPCFC in pediatric participants with single or multiple food allergies across multiple endpoints, warranting further clinical investigation.

The incidence of food allergies is rising globally, estimated to affect up to 10% of the population.^1^, ^2^, ^3^, ^4^ Reactions can be serious or life-threatening, particularly in cases of anaphylaxis. In the United States, multifood allergy is common, with approximately 30% to 60% of food-allergic patients reacting to >1 food.^2^^,^^3^^,^^5^ Multifood allergy increases the likelihood of accidental exposure, increases the challenges of allergen avoidance, and may present increased risk of nutritional deficiency.^6^, ^7^, ^8^ For patients and their families, food allergies can cause anxiety and social isolation, increase financial burden, and significantly affect quality of life.^9^, ^10^, ^11^, ^12^

Food allergy management options include dietary avoidance, education on acute treatment of allergic reactions, oral immunotherapy (OIT) for specific foods, and the anti-IgE monoclonal antibody omalizumab.^13^^,^^14^ OIT is a method of desensitization that can modulate allergic immune responses, with potential disease modifying effects;^15^, ^16^, ^17^, ^18^ it was first described for single-food allergies in 1908,^19^ with efficacy now demonstrated in larger studies.^20^, ^21^, ^22^, ^23^, ^24^, ^25^, ^26^, ^27^, ^28^, ^29^, ^30^, ^31^ Only one regulatory-approved OIT is currently available, a single-allergen treatment specifically for pediatric peanut allergy.^32^ This leaves a vast unmet need for single-allergen treatment of other prevalent food allergies, and for simultaneous treatment of multifood allergy. While studies show potential for parallel desensitization to multiple allergens using nonregulated commercial food sources as multifood OIT,^33^, ^34^, ^35^, ^36^, ^37^, ^38^ these treatments present challenges with regard to custom formulation, dose, and quality control.

ADP101 is a novel, first-in-class multifood OIT drug product, a standardized pharmaceutical-grade dry powder containing proteins from 15 common allergenic foods:^3^^,^^39^ almond, cashew, chicken’s egg, codfish, cow’s milk, hazelnut, peanut, pecan, pistachio, salmon, sesame, shrimp, soy, walnut, and wheat, in equal parts by protein weight. The Harmony trial evaluated the initial safety and efficacy of ADP101 in participants with single or multiple food allergies.

## Methods

Harmony (ClinicalTrials.gov, NCT04856865) was a multicenter, phase 1/2 randomized, double-blind, placebo-controlled trial conducted in the United States from April 2021 to December 2022, in accordance with the Declaration of Helsinki and Council for International Organizations of Medical Sciences International Ethical Guidelines, applicable International Council for Harmonisation Good Clinical Practice guidelines, and applicable laws and regulations. Participants, or their legally authorized representatives, provided written informed consent before participation. All sites were approved by their institutional review board or independent ethics committee before initiation. An independent data monitoring committee provided oversight. Alladapt Immunotherapeutics Inc sponsored the trial and was involved in its design, conduct, data collection, and analysis. CONSORT (Consolidated Standards of Reporting Trials) reporting guidelines were adhered to.^40^ The trial protocol and statistical analysis plan are available in this article’s Online Repository available at www.jaci-global.org.

### Trial population

Eligible participants were 4 to 55 years of age; however, the prespecified primary analysis population was pediatric participants (age 4-17 years). Participants had 1 to 5 qualifying food allergies (QFAs), defined as dose-limiting symptoms with a ≤100 mg challenge dose of almond, cashew, chicken’s egg, codfish, cow’s milk, hazelnut, peanut, pecan, pistachio, salmon, sesame, shrimp, soy, walnut, or wheat during double-blind, placebo-controlled food challenge (DBPCFC) at Screening. Table E1, in the Online Repository at www.jaci-global.org, lists complete enrollment criteria.

### Trial design

Eligible participants were randomized, then stratified by age (4-17 and 18-55 years, inclusive) 2:2:1:1 to ADP101 low dose (LD-ADP101; target dose 1500 mg/d [100 mg protein per food]), ADP101 high dose (HD-ADP101; target dose 4500 mg/d [300 mg protein per food]), placebo low dose, or placebo high dose. The randomization schedule was generated by an independent unblinded statistician by SAS software. Participants, assessing physicians, and participating site staff were unaware of the treatment group.

Active study drug (ADP101) was a dry powder mixture of allergenic proteins from 15 food sources (almond, cashew, chicken’s egg, codfish, cow’s milk, hazelnut, peanut, pecan, pistachio, salmon, sesame, shrimp, soy, walnut, and wheat) in equal parts by protein weight. It was formulated with excipients including an aroma/flavor masker and isomalt, then packaged into premeasured doses. ADP101 was manufactured under pharmaceutical good manufacturing practice guidelines, with appropriate control of materials and process (ie, pharmaceutical grade). The product met US Food and Drug Administration requirements for administration to participants in a clinical trial setting and was released under an investigational new drug application.

Placebo powder, provided in the same packaging as the active drug, contained excipients including an aroma/flavor masker and coloring agents to achieve similar appearance and total weight as ADP101. The study drug was mixed with a palatable, nonallergenic, age-appropriate food, taken orally daily. The maximum total volume was approximately 2 rounded tablespoons.

**Fig 1:**
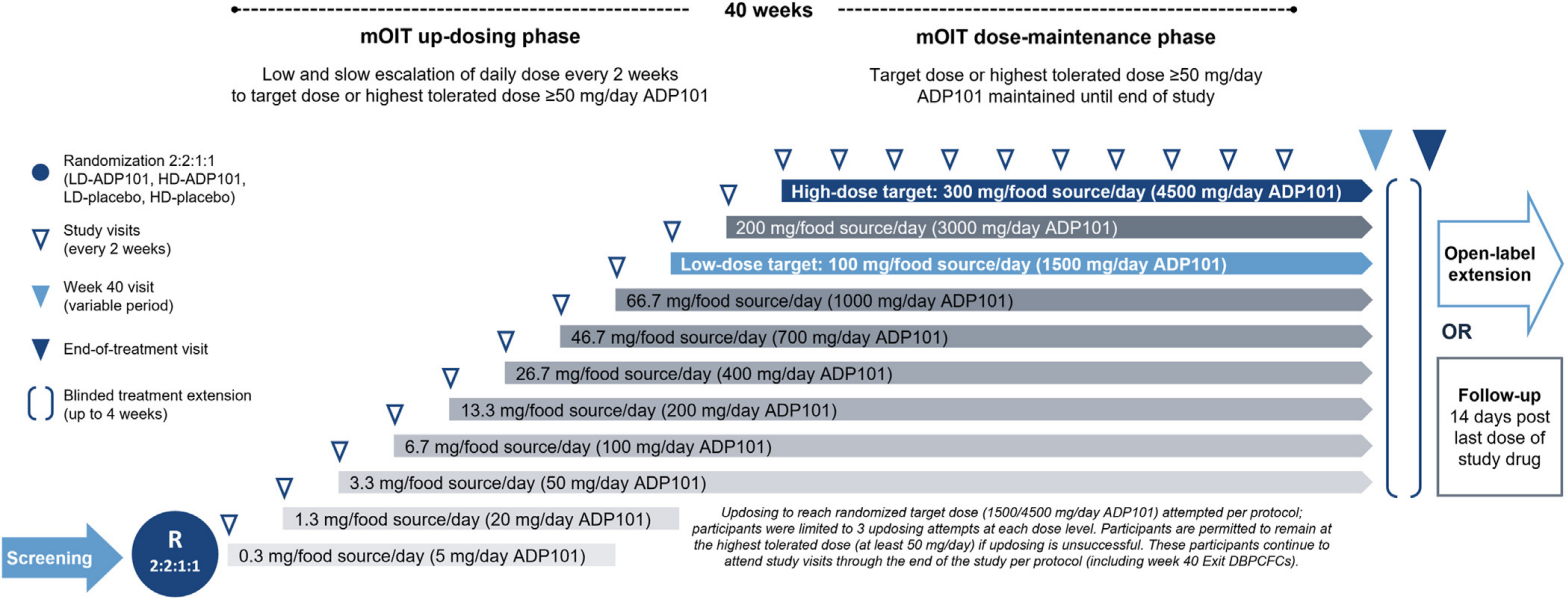
Harmony trial design and updosing schedule. Updosing was attempted every 2 weeks and permitted through Week 38. Dose (in mg) refers to protein content. *mOIT,* Multifood OIT; *R,* randomization.

In lieu of an initial dose-escalation day, Day 1 of treatment consisted of a single 5 mg dose of study drug at the trial site that, if tolerated, was continued daily at home for 2 weeks. Updosing (Fig 1) was attempted every 2 weeks in the clinic up to the randomized target dose, which was maintained through the Week 40 DBPCFCs. Patients unable to tolerate a dose level of ≥50 mg study drug were discontinued.

A key feature of the updosing protocol was flexibility. Participants could attempt updosing ≤3 times per dose level, with downdosing permitted per investigators’ clinical judgment at any time. Updosing was permitted through Week 38, after which the dose was maintained. Any participant able to sustain a maintenance dose of ≥50 mg was eligible to complete the Week 40 DBPCFCs. After the final Exit DBPCFC, all participants continued treatment for up to 4 weeks in a blinded extension period, followed by the end-of-treatment visit, to maintain blinding during subject-level data cleaning. Participants were then unblinded in a rolling order. Eligible participants who completed the study could elect to continue in the open-label extension trial (Encore; NCT05243719).

### Assessments

DBPCFCs were performed at Screening for foods with a known clinical history, significant cross-reactivity to other food allergens (eg, cashews if allergic to pistachios), or unknown reactivity if skin-prick test (SPT) mean wheal diameter was >3 mm above negative control.

DBPCFCs were performed again at Exit/Week 40 to assess desensitization to reactive foods and to assess neosensitization to foods not recently consumed but that had a ≥3 mm increase at Week 38 over Screening in SPT mean wheal diameter (relative to negative control).

The Harmony DBPCFC procedure was based on the 2012 PRACTALL consensus guidelines^41^ with modified updosing schedules (see Table E2 in the Online Repository at www.jaci-global.org). Participants were gradually fed increasing amounts of a suspected allergenic food or matched placebo, mixed with a palatable food, every 15 to 30 minutes, with 1 hour maximum between doses. The challenge was terminated if any symptom met the stopping criteria as per the study food challenge manual (ie, dose-limiting symptoms) and appropriate treatment provided. Eliciting dose (ED) for individual foods was defined as the dose level that elicited symptoms leading to DBPCFC termination. The maximum tolerated dose (MTD) was defined as the highest dose level tolerated during the DBPCFC.

DBPCFCs were performed under direct medical supervision using sponsor-provided food challenge material prepared by unblinded site staff, administered by blinded site staff/investigators. During screening, the highest single dose level per food tested in the DBPCFC was 1000 mg protein (cumulative 2044 mg). Reactive foods were those with an ED of ≤1000 mg, further categorized into qualifying foods (ED ≤100 mg) or nonqualifying foods (ED >100 mg but ≤1000 mg). During Exit DBPCFCs, the highest single dose level per food tested was 4000 mg protein (cumulative 8044 mg).

Safety assessments included adverse events (AEs), serious AEs, and AEs of special interest (AESIs). Study-defined AESIs included anaphylaxis, AEs leading to epinephrine use, and eosinophilic esophagitis (EoE). AEs were classified using the Medical Dictionary for Regulatory Activities (MedDRA) v24.1. Severity of allergic AEs, including anaphylaxis, was graded with the Consortium for Food Allergy Research (CoFAR) Grading Scale v1.^42^ Nonallergic AEs were graded with the Common Terminology Criteria for Adverse Events (CTCAE) v5.0.

### Outcomes

The primary endpoint was the proportion of participants tolerating a ≥600 mg challenge dose of a single qualifying food (1044 mg cumulative) without dose-limiting symptoms at Exit DBPCFC. Secondary endpoints were the proportion of participants tolerating ≥1000 mg of a single qualifying food, and, for participants with >1 QFA, tolerating ≥600 mg or ≥1000 mg of ≥2 qualifying foods at Exit DBPCFC. Prespecified exploratory endpoints included change from baseline in SPT and ED; MTD; maximum severity of allergy symptoms at Screening and Exit DBPCFC; incidence of neosensitization; and accidental exposure. To further evaluate ADP101 at higher levels of desensitization, additional *ad hoc* efficacy analyses were conducted to identify participants tolerating the ≥2000 or 4000 mg challenge dose of a qualifying food at Exit DBPCFC.

### Statistical analysis

The primary analysis population was the pediatric intent-to-treat (ITT) population, defined as all randomized participants aged 4 to 17 years, inclusive. The planned primary analysis population was 60 participants (20 in each of the HD-ADP101, LD-ADP101, and placebo groups). Adequacy of the sample size was confirmed by performing simulations for power calculations by Fisher exact test for each of 2 doses (vs placebo) under alternative hypotheses. Assuming placebo response rates of 4% and active response rates of 40% and 58% in the low- and high-dose regimen treatment groups, respectively (based on Vickery et al^29^) and an alpha of 5%, power was calculated by the Simes global test^43^ with the Holm procedure^44^ to reject ≥1 of the doses. With a 20% dropout rate, results showed approximately 90% power to detect a statistically significant ADP101 response in ≥1 of the doses with the given sample size.

Unless otherwise specified, information was summarized descriptively by treatment arm, including number of patients, mean, standard deviation (SD), median, range for continuous variables, and number and percentage of nonmissing values per category for categorical data. Low- and high-dose placebo groups were pooled for analysis. The primary and secondary endpoints were binary data, and respective difference in the proportion of responders (those tolerating a ≥600 mg challenge dose of ≥1 qualifying food at Exit DBPCFC; ADP101 vs pooled placebo) with Newcombe 95% confidence intervals were reported together with the unadjusted *P* values by Fisher exact test. Participants missing Exit DBPCFC data were considered nonresponders unless otherwise specified. In the ITT analysis, to adjust for multiple comparisons for the primary endpoint, the Simes test with the Holm procedure was applied, comparing the lowest unadjusted *P* value against an adjusted threshold of 0.025 to test for statistical significance. In the supplemental ITT analysis, response status was imputed by multiple imputation with logistic regression for participants discontinuing for administrative reasons (eg, site closure); all other discontinuations were considered nonresponse. The logistic regression model included treatment group, number of qualifying food allergies, median specific IgE-to-IgG_4_ ratio for qualifying foods at baseline, and history of asthma as covariates. Both adjusted and unadjusted *P* values are reported for the primary endpoint; subsequent analyses report the unadjusted (nominal) *P* value only. Analysis was performed by SAS v9.4 or higher.

## Results

### Participants

**Fig 2:**
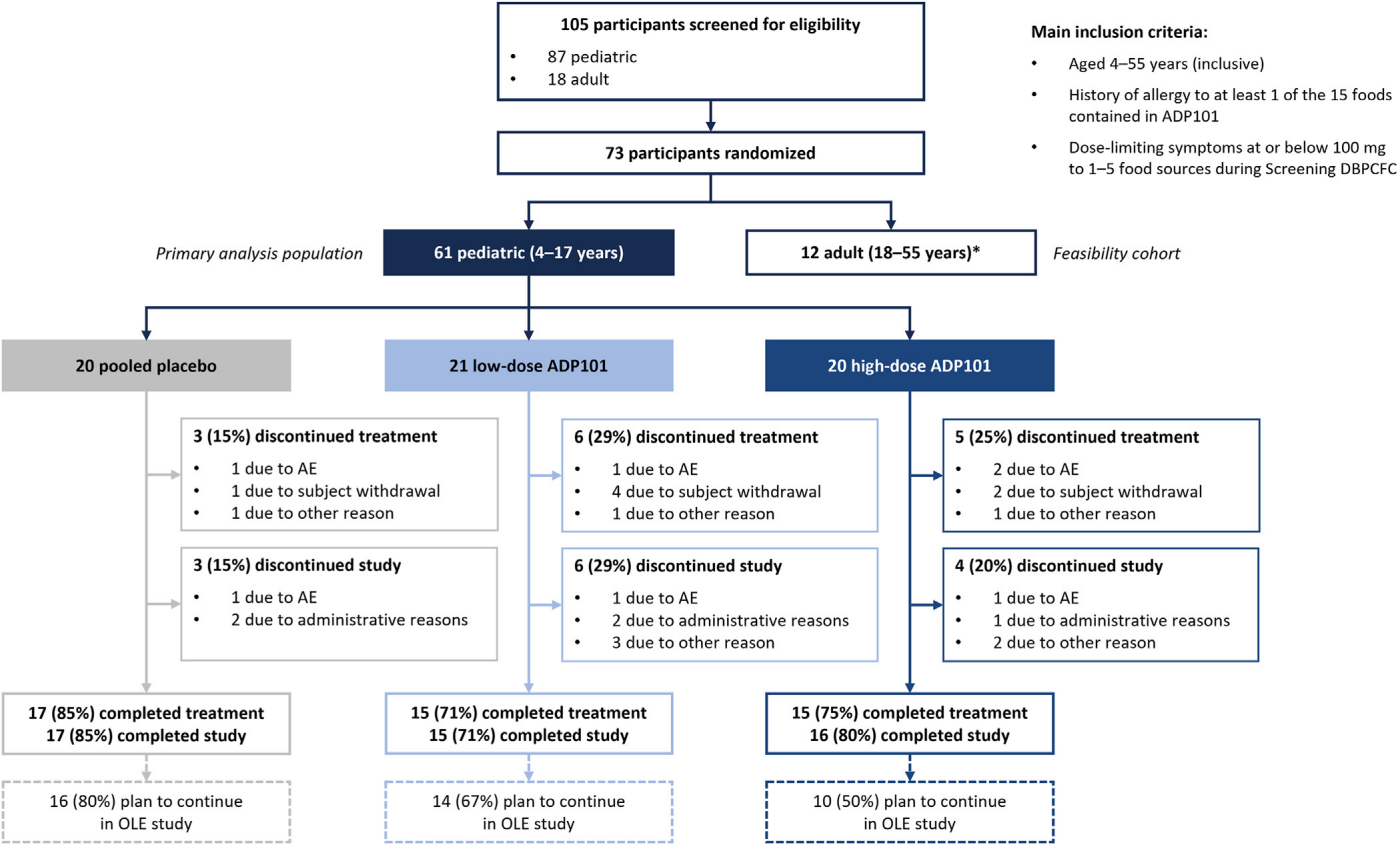
Participant screening, randomization, and follow-up. Of 105 participants, 32 (31%) did not meet inclusion criteria (17%), had exclusion criteria (4%), or had another reason (10%) to be removed from study; other reasons included withdrawal of consent, time constraints, planned travel, investigator recommendation, SPT reaction not within protocol limits, and SPT-negative control reaction >3 mm. Primary analysis population (N = 61) was the pediatric ITT population. In this group, 47 (77%) of 61 completed treatment, with 40 (66%) planning to continue in the OLE. Treatment discontinuation occurred in 14 (23%) of pediatric participants as a result of patient withdrawal (12%), AEs (7%), and other reasons (5%); other reasons included site closure/declined site transfer. One participant who discontinued treatment continued study participation through the end-of-treatment visit. All other participants who discontinued treatment also discontinued the study. Thirteen (21%) of 61 discontinued study, with primary reason being administration (8%), other (8%), and AE (5%); other reasons included withdrawal of consent because of product taste and/or texture, concerns over number of food challenges and school time missed, noncompliance, and inability to attend study visits. ∗Twelve adult participants were included as a feasibility cohort; cases are described individually in Online Repository at www.jaci-global.org. *OLE,* Open-label extension.

A total of 73 participants were randomized, including 61 pediatric and 12 adult participants (Fig 2) from 15 sites. Results presented here are for the study-defined primary analysis population (pediatric ITT population); results for adult participants are provided in Fig E1 and Table E3 in the Online Repository at www.jaci-global.org.

In the pediatric ITT population, the median (range) age at Screening was 10 (4-17) years (Table I). QFAs to 14 of 15 components of ADP101 were represented in the pediatric ITT; moreover, 49.2% of participants had ≥2 QFAs, and 45.9% of participants had reactive nonqualifying food allergies in addition to ≥1 QFA (see Table E4 in the Online Repository at www.jaci-global.org). The majority of participants had a history of anaphylaxis. Peanut, followed by cashew, were the most common QFAs. Multifood allergy was higher in the placebo group than in the active treatment groups; 60.0% (12/20), 42.9% (9/21), and 45.0% (9/20) of participants had ≥2 QFAs in the placebo, LD-ADP101, and HD-ADP101 groups, respectively.

**Table I:** Baseline characteristics of pediatric ITT population

Characteristic	Pooled placebo (n = 20)	LD-ADP101 (n = 21)	HD-ADP101 (n = 20)
Age at Screening (years)			
Mean [SD]	10.1 [3.5]	10.3 [3.4]	9.9 [2.8]
Median (range)	9.5 (4-16)	10.0 (5-17)	10.0 (5-14)
Sex, no. (%)			
Male	13 (65.0)	14 (66.7)	14 (70)
Female	7 (35.0)	7 (33.3)	6 (30.0)
Race, no. (%)			
White	10 (50.0)	15 (71.4)	16 (80.0)
Asian	5 (25.0)	5 (23.8)	2 (10.0)
Black or African American	3 (15.0)	0	0
Other	2 (10.0)	1 (4.8)	2 (10.0)
Total IgE at Screening (IU/mL)			
Mean [SD]	559.5 [978.4]	650.6 [597.6]	811.4 [1567.3]
Median (range)	270.4 (28.4-4486.4)	372.3 (7.5-2111.8)	350.6 (34.5-6824.3)
**Qualifying reactive foods**			
Food allergies per participant based on Screening DBPCFC, no. (%)			
1	8 (40.0)	12 (57.1)	11 (55.0)
2	5 (25.0)	4 (19.0)	3 (15.0)
3	3 (15.0)	4 (19.0)	2 (10.0)
4	2 (10.0)	0	2 (10.0)
5	2 (10.0)	1 (4.8)	2 (10.0)
Individual food allergies based on Screening DBPCFC, no. (%)			
Peanut	11 (55.0)	9 (42.9)	12 (60.0)
Cashew	10 (50.0)	7 (33.3)	5 (25.0)
Pistachio	8 (40.0)	4 (19.0)	4 (20.0)
Walnut	5 (25.0)	3 (14.3)	3 (15.0)
Chicken’s egg	1 (5.0)	5 (23.8)	3 (15.0)
Pecan	3 (15.0)	3 (14.3)	2 (10.0)
Cow’s milk	3 (15.0)	3 (14.3)	1 (5.0)
Hazelnut	3 (15.0)	0	3 (15.0)
Sesame seed	0	2 (9.5)	1 (5.0)
Almond	1 (5.0)	0	1 (5.0)
Codfish	0	0	2 (10.0)
Shrimp	0	0	2 (10.0)
Wheat	0	0	2 (10.0)
Salmon	0	1 (4.8)	0
Soy	0	0	0
Baseline SPT (mm), mean [SD]			
Almond	12.5 [NA]		8.0 [NA]
Cashew	12.3 [4.8]	19.7 [11.1]	19.1 [10.1]
Chicken’s egg	16.5 [NA]	13.1 [12.0]	8.2 [8.5]
Codfish			3.8 [5.3]
Cow’s milk	25.7 [24.8]	12.7 [9.1]	16.0 [NA]
Hazelnut	8.3 [5.8]		13.8 [14.0]
Peanut	9.9 [4.3]	13.4 [7.7]	15.7 [7.7]
Pecan	4.5 [7.8]	0.0 [0.0]	0.0 [0.0]
Pistachio	13.0 [6.6]	23.6 [4.9]	20.4 [8.1]
Salmon		7.0 [NA]	
Sesame seed		12.5 [4.2]	7.5 [NA]
Shrimp			13.3 [8.8]
Soy			
Walnut	14.1 [7.6]	8.0 [2.5]	13.0 [10.6]
Wheat			4.9 [0.2]
Baseline sIgE (kU_A_/L), mean [SD]			
Almond	14.1 [NA]		15.7 [NA]
Cashew	5.6 [5.1]	23.0 [32.5]	6.3 [9.1]
Chicken’s egg	37.0 [NA]	23.4 [31.7]	1.3 [1.2]
Codfish			0.1 [NA]
Cow’s milk	25.5 [34.8]	40.7 [51.7]	100.0 [NA]
Hazelnut	3.9 [2.9]		14.4 [14.9]
Peanut	39.4 [48.1]	53.0 [38.7]	32.5 [35.2]
Pecan	3.5 [2.4]	34.1 [14.5]	6.6 [9.1]
Pistachio	9.0 [7.7]	40.1 [41.1]	35.7 [44.0]
Salmon		0.1 [NA]	
Sesame seed		30.7 [37.3]	36.5 [NA]
Shrimp			0.7 [0.8]
Soy			
Walnut	9.5 [11.3]	60.1 [49.1]	12.4 [12.3]
Wheat			86.0 [19.8]
Medical history, no. (%)			
Anaphylaxis	16 [80.0]	17 [81.0]	10 [50.0]
Asthma	8 [40.0]	8 [38.1]	8 [40.0]
Eczema	4 [20.0]	5 [23.8]	3 [15.0]

Qualifying reactive food defined as food eliciting reaction at ≤100 mg during Screening DBPCFC.

*NA,* Not applicable; *sIgE,* specific IgE.

A total of 78.7% (48/61) of participants completed the trial, with 77.0% (47/61) completing treatment. A total of 21.3% (13/61) discontinued the trial (Fig 2); 1.6% (1/61) discontinued the study drug because of an AE but completed the trial. One site closed because of coronavirus disease 2019 (COVID-19)-related administrative reasons; 3 of 4 participants at this site discontinued the trial, and 1 transferred to another site and completed the trial.

### Treatment and exposures

Overall treatment compliance in the pediatric ITT population was >90% (see Table E5 in the Online Repository at www.jaci-global.org). The target treatment dose was achieved in 90.0%, 76.2%, and 70.0% of placebo, LD-ADP101, and HD-ADP101 participants, respectively, over a median of 20.6, 20.6, and 24.2 weeks of updosing. Median time at maintenance dose was 142.0, 145.0, and 112.0 days in the placebo, LD-ADP101, and HD-ADP101 group, respectively.

Median (range) maintenance dose was 4500 (1500-4500) mg, 1500 (50-1500) mg, and 4500 (50-4500) mg in the placebo, LD-ADP101, and HD-ADP101 group, respectively. A total of 90.0% of pooled placebo and 75.6% of pooled ADP101 participants reached at least 1500 mg as their highest tolerated dose, with 100.0% of high-dose placebo and 65.0% of HD-ADP101 participants reaching 4500 mg as their highest tolerated dose.

### Primary endpoint

**Fig 3:**
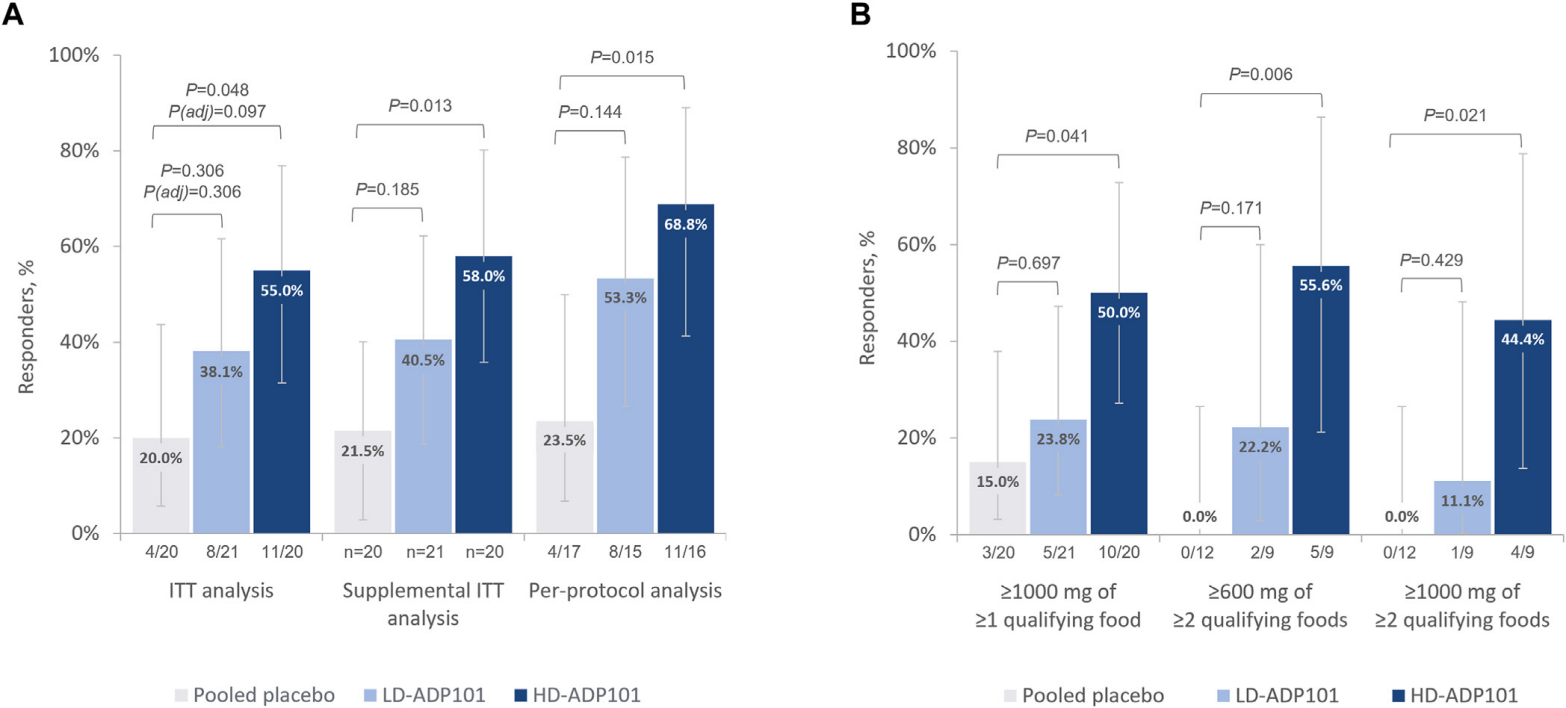
Response rates for primary and secondary efficacy endpoints (pediatric participants). **(A)** Primary efficacy endpoint; participants tolerating ≥600 mg of ≥1 qualifying food at Exit DBPCFC. ITT population was defined as all randomized participants. The primary endpoint was not met after adjustment for multiple comparisons. In supplemental ITT analysis, response status was imputed using multiple imputation for participants discontinuing for administrative reasons; all other discontinuations were considered nonresponse. Per-protocol population is defined as all participants from ITT population without major protocol deviations that affect statistical analysis, conducted as a sensitivity analysis of primary efficacy endpoint. **(B)** Secondary efficacy endpoints. *P* values are unadjusted unless otherwise stated, determined by Fisher exact test. Adjusted *P* values were determined by Simes global test with Holm procedure. Error bars depict 95% CI of proportion, estimated by Clopper-Pearson exact method. Dose (in mg) refers to protein content. *Adj,* Adjusted; *CI,* confidence interval.

The proportion of pediatric ITT participants tolerating a ≥600 mg challenge dose of ≥1 qualifying food at Exit DBPCFC (ie, responders) was 20.0% (4/20), 38.1% (8/21), and 55.0% (11/20) for placebo, LD-ADP101, and HD-ADP101 participants, respectively (unadjusted *P* values vs placebo: LD-ADP101, *P* = .306; HD-ADP101, *P* = .048) (Fig 3, *A*). The trial did not meet its primary endpoint after adjustments for multiple comparisons (adjusted *P* values vs placebo: LD-ADP101, *P* = .306; HD-ADP101, *P* = .097).

To accommodate challenges of the COVID-19 pandemic, a prespecified supplemental ITT analysis was performed in which responses were imputed by treatment group for participants who discontinued for reasons unrelated to study drug (eg, COVID-19–related site closure). Using this hybrid estimand strategy, 21.5% placebo, 40.5% LD-ADP101, and 58.0% HD-ADP101 participants were responders (nominal *P* values vs placebo: LD-ADP101, *P* = .185; HD-ADP101, *P* = .013).

In the pediatric per-protocol population (including only participants who completed Exit DBPCFC), 23.5%, 53.3%, and 68.8% of placebo, LD-ADP101, and HD-ADP101 participants, respectively, were responders (nominal *P* values vs placebo: LD-ADP101, *P* = .144; HD-ADP101, *P* = .015).

### Secondary endpoints

A total of 15.0%, 23.8%, and 50% of placebo, LD-ADP101, and HD-ADP101 participants tolerated a ≥1000 mg challenge dose of ≥1 qualifying food at Exit DBPCFC (Fig 3, *B*). Among participants with multiple food allergies (>1 QFA) in the placebo (n = 12), LD-ADP101 (n = 9), and HD-ADP101 (n = 9) groups, respectively, 0, 22.2%, and 55.6% tolerated a ≥600 mg challenge dose, and 0, 11.1%, and 44.4% tolerated a ≥1000 mg challenge dose of ≥2 qualifying foods.

### ADP101 dose threshold analysis

**Fig 4:**
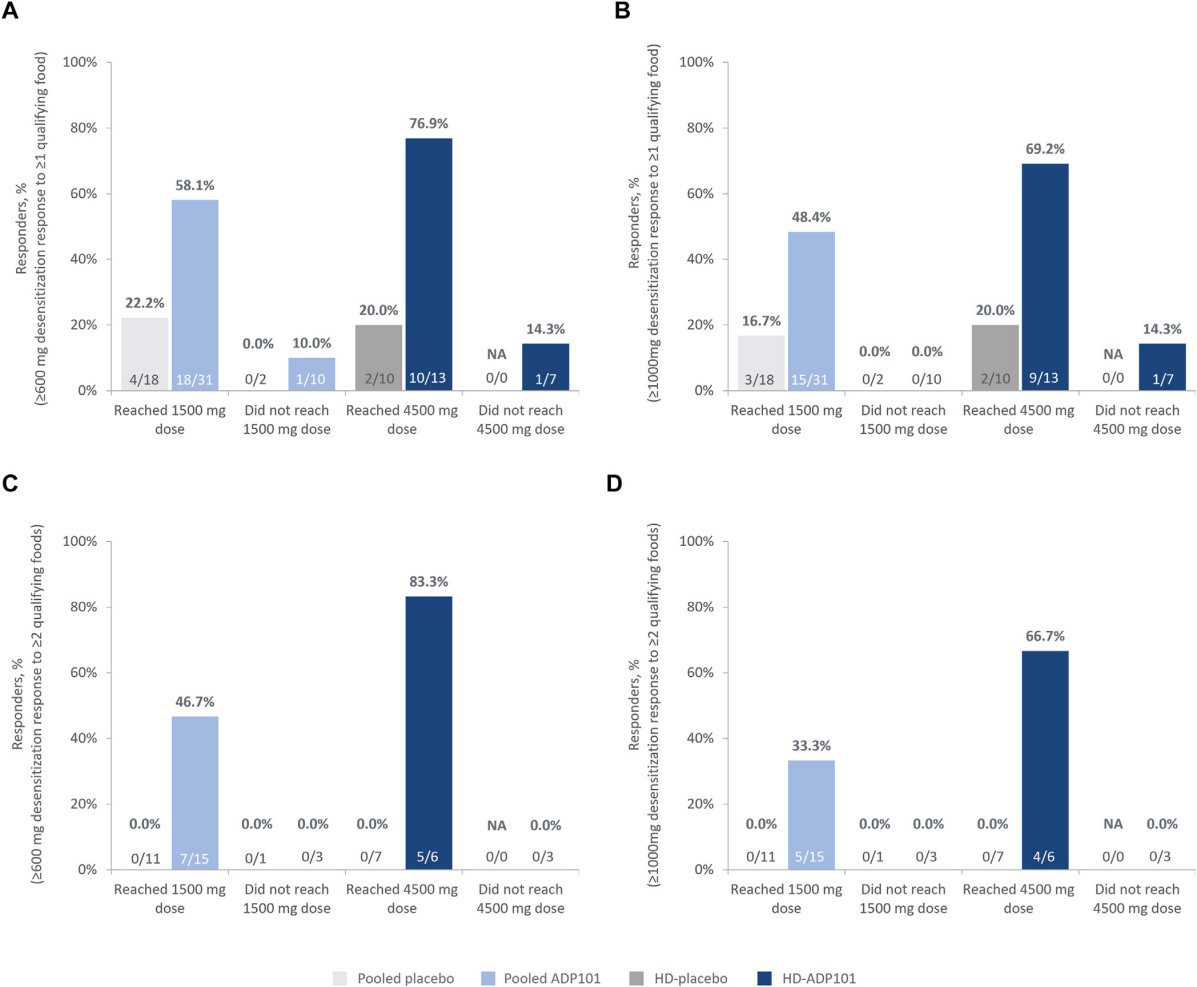
Response rate in participants reaching or not reaching 1500 mg or 4500 mg of study drug (pediatric ITT population). ADP101 dose threshold analysis. Patients randomized to both LD-ADP101 and HD-ADP101 could reach 1500 mg dose (pooled ADP101), whereas only those randomized to HD-ADP101 could reach 4500 mg dose. Reached dose refers to participants who were treated with specified dose and tolerated this for ≥2 weeks. **(A)** Participants with a ≥600 mg desensitization response to ≥1 qualifying food at exit DBPCFC. **(B)** Participants with a ≥1000 mg desensitization response to ≥1 qualifying food at exit DBPCFC. **(C)** Participants with a ≥600 mg desensitization response to ≥2 qualifying foods at exit DBPCFC. **(D)** Participants with a ≥1000 mg desensitization response to ≥2 qualifying foods at exit DBPCFC. *NA,* Not applicable.

Rates of desensitization at the 600 mg or 1000 mg threshold to ≥1 food were shown to be very low for participants unable to tolerate a ≥1500 mg (100 mg protein per food) maintenance dose (0 for pooled placebo; ≤10% response for pooled ADP101) (Fig 4). However, among LD-ADP101 and HD-ADP101 (pooled ADP101) participants who could tolerate a ≥1500 mg maintenance dose, response rates increased to 58.1% (600 mg threshold) and 48.4% (1000 mg threshold). Among HD-ADP101 patients who reached the 4500 mg target maintenance dose (300 mg protein per food), 76.9% and 69.2% had 600 mg and 1000 mg desensitization thresholds, respectively. Similar results were observed in participants with multiple food allergies for desensitization to ≥2 qualifying foods (Fig 4, *C* and *D*). This ADP101 dose threshold analysis was modified from that specified in the statistical analysis plan, including both LD-ADP101 and HD-ADP101 patients in the 1500 mg analyses as both groups provided the opportunity to achieve this dose.

### Subgroup analysis

Individual food results were consolidated into 9 categories,^39^ grouping tree nuts and finfish (see Table E6 in the Online Repository at www.jaci-global.org). Four categories included ≥1 participant from each treatment group: peanut, tree nut, chicken’s egg, and cow’s milk. Rates of tolerance to a ≥600 mg challenge dose in the placebo, LD-ADP101, and HD-ADP101 treatment groups, respectively, were for peanut 9.1%, 11.1%, and 41.7%; tree nut 21.4%, 44.4%, and 71.4%; chicken’s egg 0, 20.0%, and 0; and cow’s milk 0, 66.7%, and 100.0%.

### *Ad hoc* analyses and exploratory endpoints

**Fig 5:**
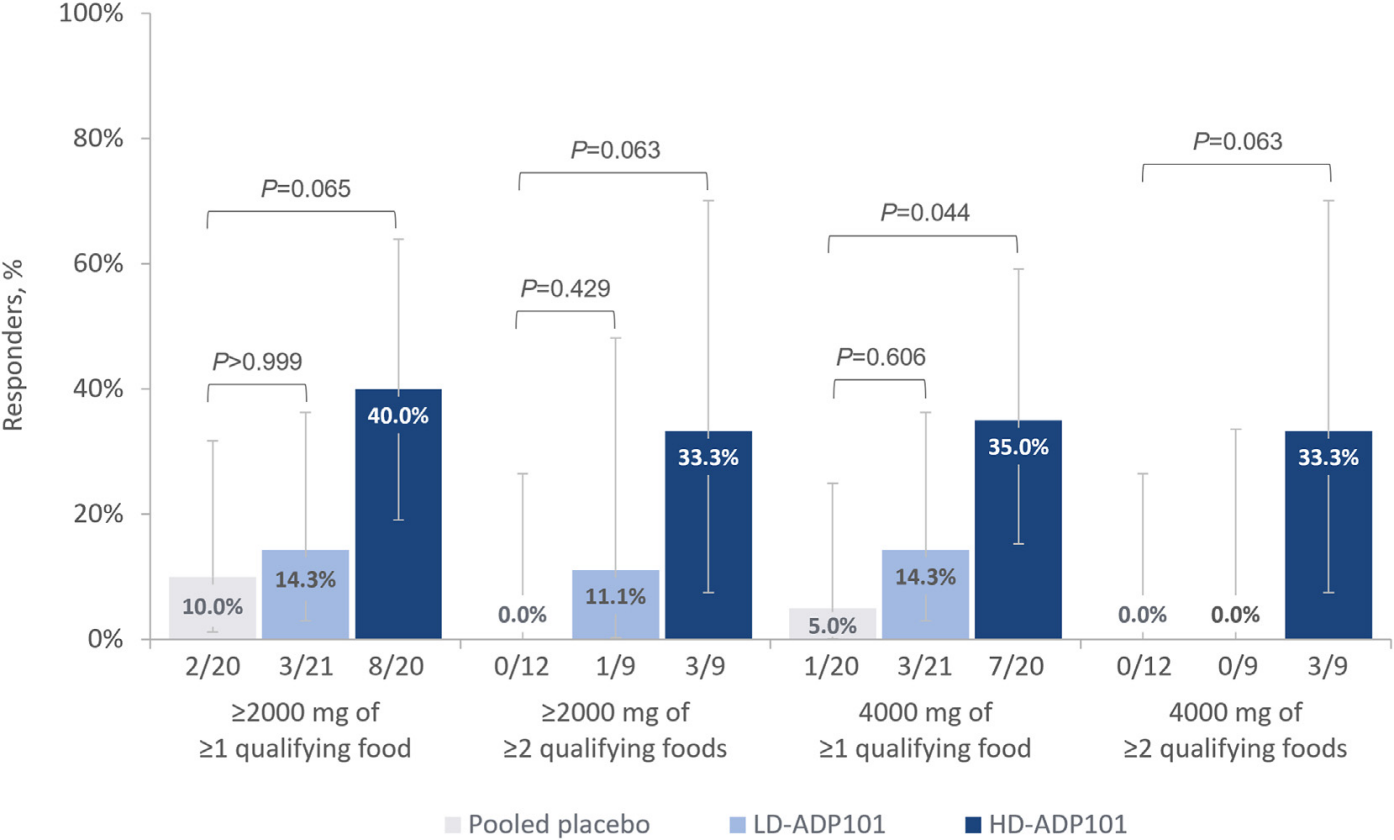
Participants tolerating higher levels (≥2000 mg) of ≥1 qualifying food (pediatric ITT population). *Ad hoc* efficacy analysis (not included in statistical analysis plan). Desensitization response defined as tolerating ≥2000 mg or 4000 mg challenge dose without dose-limiting symptoms at Exit DBPCFC. All *P* values are unadjusted, determined by Fisher exact test. Error bars depict 95% CI of proportion, estimated by Clopper-Pearson exact method. *CI,* Confidence interval.

In *ad hoc* analyses evaluating efficacy of ADP101 at higher levels of desensitization (Fig 5), the proportion of participants in the placebo, LD-ADP101 and HD-ADP101 treatment groups, respectively, tolerating the ≥2000 mg challenge dose of ≥1 qualifying food were 10.0%, 14.3%, and 40.0%; and of ≥2 qualifying foods were 0, 11.1%, and 33.3%. Rates of tolerance to a ≥4000 mg challenge dose of ≥1 qualifying food were 5.0%, 14.3%, and 35.0%; and of ≥2 qualifying foods 0, 0, and 33.3%.

Results for prespecified exploratory endpoints, including change from baseline in SPT, MTD, and ED by individual food; maximum severity of allergy symptoms at DBPCFC; neosensitization; and accidental exposure, are included in the Online Repository at www.jaci-global.org in Figs E2 and E3, and Tables E7, E8, and E9. ADP101-treated participants showed an overall reduction in SPT reactivity (Fig E2), with an increase in MTD across the majority of foods tested (Fig E3 and Table E7).

### Safety

A small proportion of participants experienced AEs on day 1, including 2 placebo-, 2 LD-ADP101–, and 2 HD-ADP101–treated participants (see Table E10 in the Online Repository at www.jaci-global.org). These were mild or moderate in severity and did not result in study discontinuation.

Most participants had ≥1 treatment-emergent AE (TEAE) over the treatment period, including 95.0% placebo-, 95.2% LD-ADP101–, and 95.0% HD-ADP101–treated participants (Table II, and see Table E11 in the Online Repository at www.jaci-global.org). The rate of TEAEs per dose was 5.4%, 6.0%, and 5.9% in the placebo, LD-ADP101, and HD-ADP101 groups, respectively. The majority of TEAEs were mild or moderate, occurring more frequently during updosing (Table E10).

**Table II:** Overall summary of TEAEs in pediatric participants

Category	Pooled placebo (n = 20)	LD-ADP101 (n = 21)	HD-ADP101 (n = 20)
Total number of TEAEs	295	327	318
Participants with any TEAE	19 (95.0)	20 (95.2)	19 (95.0)
Participants with TEAE related to study drug	12 (60.0)	16 (76.2)	15 (75.0)
Participants with TEAE due to accidental exposure	3 (15.0)	1 (4.8)	5 (25.0)
Participants with TEAE due to Exit DBPCFC	16 (80.0)	15 (71.4)	13 (65.0)
Participants with TEAE due to other trial procedure	0	0	0
Participants with TEAE resulting in drug discontinuation	1 (5.0)	1 (4.8)	2 (10.0)
Participants with TEAE resulting in drug interruption	9 (45.0)	9 (42.9)	10 (50.0)
Participants with TEAE resulting in dose modification	6 (30.0)	9 (42.9)	9 (45.0)
Participants with TEAE resulting in trial discontinuation	1 (5.0)	1 (4.8)	1 (5.0)
Participants with TEAE with fatal outcome	0	0	0
Allergic/nonallergic TEAEs			
Participants with allergic TEAE	17 (85.0)	20 (95.2)	16 (80.0)
Participants with CoFAR grade 3 or higher allergic TEAE	3 (15.0)	2 (9.5)	2 (10.0)
Participants with CoFAR grade 4 or higher allergic TEAE	0	0	0
Participants with CTCAE grade 3 or higher nonallergic TEAE	0	0	0
Participants with CTCAE grade 4 or higher nonallergic TEAE	0	0	0
TESAEs			
Participants with TESAE	0	0	1 (5.0)
Participants with TESAE related to trial drug	0	0	0
Participants with TESAE due to accidental exposure	0	0	0
Participants with TESAE due to Exit DBPCFC	0	0	0
Participants with TESAE due to other trial procedure	0	0	0
AESIs			
Participants with AESI	11 (55.0)	9 (42.9)	10 (50.0)

Data are presented as nos. (%). Allergic TEAEs were graded based on CoFAR Grading Scale v1; nonallergic TEAEs were graded based on National Cancer Institute CTCAE v5.0. *CoFAR,* Consortium of Food Allergy Research; *CTCAE,* Common Terminology Criteria for Adverse Events; *TESAE,* serious TEAE.

Four of 61 participants discontinued the study drug as a result of AEs, 3 of whom entirely discontinued the trial (1 each in the placebo [anaphylaxis], LD-ADP101 [EoE], and HD-ADP101 [anxiety] groups). One participant in the HD-ADP101 group discontinued treatment because of dyspepsia but completed the trial.

The majority of participants in the safety population experienced ≥1 study drug-related TEAE—60.0%, 76.2%, and 75.0% of the placebo, LD-ADP101, and HD-ADP101 groups, respectively (Table II). During dose maintenance, no TEAEs related to study drug occurred in >10% of any group, and there were no study drug-related TEAEs of anaphylaxis (see Table E12 in the Online Repository at www.jaci-global.org).

Overall rates of treatment-emergent AESI (including Exit DBPCFC) were balanced between groups, occurring in 55.0%, 42.9%, and 50.0% of placebo-, LD-ADP101–, and HD-ADP101–treated participants, respectively (Table II). One biopsy-confirmed case of EoE occurred in the LD-ADP101 group during updosing; this resolved (symptomatically and histologically) after treatment discontinuation. TEAEs leading to epinephrine use, and of anaphylaxis, were more frequent during updosing; these were primarily mild or moderate (Table III). Overall, grade 3 anaphylaxis was infrequent, and no grade 3 events were attributed to ADP101. No grade 4 anaphylaxis was reported.

**Table III:** AESIs in pediatric safety population

Category	Updosing phase			Dose-maintenance phase		
	Pooled placebo (n = 20)	LD-ADP101 (n = 21)	HD-ADP101 (n = 20)	Pooled placebo (n = 20)	LD-ADP101 (n = 21)	HD-ADP101 (n = 20)
Participants with ≥1 AESI	3 (15.0)	5 (23.8)	7 (35.0)	1 (5.0)	0	1 (5.0)
Participants with ≥1 anaphylaxis event	3 (15.0)	4 (19.0)	6 (30.0)	1 (5.0)	0	1 (5.0)
Grade 3 or higher (severe)	1 (5.0)	0	1 (5.0)	0	0	0
Grade 4 or higher (life-threatening)	0	0	0	0	0	0
Participants with ≥1 epinephrine use	1 (5.0)	3 (14.3)	7 (35.0)	1 (5.0)	0	1 (5.0)
AE prompting epinephrine use	1 (5.0)	3 (14.3)	7 (35.0)	1 (5.0)	0	1 (5.0)
Related to study drug	1 (5.0)	2 (9.5)	5 (25.0)	0	0	0
Accidental exposure	0	0	0	1 (5.0)	0	1 (5.0)
Other	0	1 (4.8)	2 (10.0)	0	0	0
Severity of TEAE prompting epinephrine use						
Grade 1 (mild)	0	0	4 (20.0)	0	0	0
Grade 2 (moderate)	0	3 (14.3)	2 (10.0)	1 (5.0)	0	1 (5.0)
Grade 3 (severe)	1 (5.0)	0	1 (5.0)∗	0	0	0
Grade 4 (life-threatening)	0	0	0	0	0	0
Grade 5 (death)	0	0	0	0	0	0
Participants with ≥1 EoE event	0	1 (4.8)†	0	0	0	0

Data are presented as nos. (%). Anaphylaxis grading based on Consortium of Food Allergy Research (CoFAR) Grading Scale v1. Participants who experienced multiple events within AESI type were counted once for that AESI type.

∗ Cause unknown; occurred during early weeks of updosing before desensitization might be expected and in setting of potential accidental exposure to allergen. This event was considered serious because it required hospitalization for observation.

† Biopsy-confirmed EoE occurring approximately 4 weeks after reaching target dose of 1500 mg; participant discontinued treatment, and disease showed histologic resolution at repeat endoscopy.

There was 1 serious AE (anaphylaxis; Table III) reported, and no life-threatening TEAEs or deaths (Table II).

## Discussion

ADP101 is a novel investigational multifood OIT that includes 15 foods, which are representative of the food groups implicated in >90% of food allergies.^39^ While the primary endpoint was not met in the Harmony trial, ADP101 increased the reactive threshold in pediatric participants with single and multiple food allergies, with increased response rate at a higher dose of ADP101 observed across sensitivity analyses of the primary endpoint and by multiple prespecified subgroup and exploratory analyses. Evidence of desensitization was observed across most individual foods in ADP101-treated participants.

The 600 mg desensitization threshold for the primary efficacy endpoint is clinically meaningful and represents a ≥6-fold increase in the MTD. For peanut-allergic patients, increasing the threshold from ≤100 mg protein to 300 mg reduces the risk of allergic reaction by >95% in the event of trace contamination.^45^ An increase to 600 mg, used as the primary endpoint for PALISADE (peanut OIT)^29^ and Harmony, has the potential to confer additional risk reduction. In this trial, desensitization to ≥600 mg of peanut protein was observed in 41.7% of allergic HD-ADP101–treated participants who completed Exit DBPCFC (9.1% and 11.1% in placebo- and LD-ADP101–treated participants, respectively). Cashew was the second most prevalent allergy, with 50.0% of HD-ADP101–treated allergic participants who completed Exit DBPCFC experiencing tolerance to ≥600 mg (11.1% and 60.0% in placebo- and LD-ADP101–treated participants, respectively).

In prespecified subgroup analyses, response rate was enhanced in ADP101-treated participants who could tolerate 1500 mg/d (100 mg protein per food), and further in those who could tolerate 4500 mg/d (300 mg protein per food); 77% (10/13) of participants tolerating 4500 mg/d had disease that responded to therapy. This highlights 300 mg protein per food as the most likely effective dose for desensitization. Combined with similar rates of treatment-related AEs in LD-ADP101 and HD-ADP101 treatment groups, this supports further study of ADP101 with 4500 mg/d as the target dose, potentially with a longer treatment duration.

Evidence for desensitization at higher challenge dose levels (2000 mg, 4000 mg) was observed in *ad hoc* analyses, with desensitization to 4000 mg of ≥2 qualifying foods observed in >30% of HD-ADP101 participants with multiple food allergies. Within the limitations of small study size, this observation is encouraging; this level of oral tolerance far exceeds currently approved standards for desensitization in OIT clinical trials.^29^^,^^46^ For example, a 4000 mg challenge dose of peanut is equivalent to approximately 16 peanuts (see Table E13 in the Online Repository at www.jaci-global.org)—a potentially life-changing improvement from a baseline MTD of 3 mg (∼1/80 of a single peanut).^47^

Although TEAEs were comparable across all 3 treatment groups, and those related to study drug were comparable between LD-ADP101 and HD-ADP101, suggestive of no dose-dependent safety signals, there were more AESIs attributable to HD-ADP101 in the updosing phase. Overall, ADP101 demonstrated a safety profile expected for an OIT product.^29^ The majority of AEs were mild or moderate in severity; they were most frequently reported during updosing, with decreased frequency during dose maintenance. In contrast to other OIT trials that require an initial dose-escalation day,^20^^,^^25^^,^^29^^,^^42^ all Harmony participants received the same single starting dose of 5 mg (0.33 mg protein per food in ADP101) on Day 1, which was continued daily for 2 weeks. AE rates are typically high during a dose-escalation day; however, in Harmony, Day 1 AEs were infrequent. The patient- and provider-centric low-and-slow approach was devised to improve tolerability and reduce clinical burden, with no apparent loss of efficacy. Notably, there was no difference in the rate of neosensitization between placebo- and ADP101-treated participants, with one instance each in the placebo and LD-ADP101 groups (both hazelnut; see the Online Repository at www.jaci-global.org), suggesting that dosing with multiple allergenic foods does not pose a risk of sensitization in those who are not allergic to them.

Small sample size was a limitation of this study, contributing to variation in some baseline characteristics across the 3 groups. In addition, variable prevalence of individual allergies within each group precluded comparisons between ADP101 and placebo for several foods. The estimated sample size (N = 60) was based on placebo response rate for the approved single food (peanut) OIT, which was very low (5/124, 4%).^29^^,^^32^ Our study’s placebo response rate was higher than anticipated (4/21, 21%), the result of 4 participants tolerating a ≥600 mg challenge dose of 1 qualifying food at Exit DBPCFC (peanut, pistachio, cashew, or hazelnut). Spontaneous response was observed to be more likely in those with single allergies—notably, no participant in the placebo group with multifood allergy could tolerate ≥600 mg or ≥1000 mg of ≥2 qualifying foods at Exit DBPCFC.

Beyond approved peanut monotherapy,^32^ patients with food allergy have limited options for OIT, including nonregulated commercial food sources, with their protein content varying in unknown ways. ADP101, with a precise mixture of pharmaceutical-grade allergenic proteins from 15 food sources, has the potential to offer a standardized OIT option for those with a range of prevalent single food allergies, or with multifood allergy, which affects up to 60% of food-allergic patients in the United States.^2^^,^^3^^,^^5^ In contrast to existing options, a precisely characterized, pharmaceutical-grade comprehensive OIT such as ADP101 would ensure consistent quality, enable straightforward prescribing and pharmacy stocking, and potentially ease the burden on clinicians and multiallergic families. The approval of the monoclonal antibody omalizumab for those with multifood allergy^13^^,^^14^ represents important progress in the field; however, accompanying limitations, including the need for regular injections and unclear accessibility as a result of cost, mean that further options are required to address the diverse needs of the food allergy population. Further, anti-IgE therapy and OIT have the potential to be combined in a synergistic approach.

In conclusion, ADP101 increased the reactive threshold in pediatric participants with single or multiple food allergies, with a favorable safety profile. The trial did not meet its primary endpoint, but the data offer important insights that warrant further clinical investigation of ADP101.

**Clinical implication**

ADP101, an OIT candidate covering the 15 most prevalent food allergens, was well tolerated and increased the reactive threshold in pediatric participants with single or multiple food allergies, warranting its further investigation.

## Disclosure statement

The Harmony trial was funded by Alladapt Immunotherapeutics Inc.

Disclosure of potential conflict of interest: E. Kim is a consultant for ALK-Abello, Belhaven BioPharma, Cellergy Pharma, DBV Technologies, Genentech, Hanimune Therapeutics, Kenota Health, Novartis, 10.13039/501100001720Nutricia Research Foundation, Phylaxis, Revolo Biotherapeutics, and Ukko; and receives grant funding to his institution from the National Institute of Allergy and Infectious Diseases and 10.13039/100006423Food Allergy Research and Education (FARE). W. Carr is an employee of Allergy & Asthma Associates of Southern California; a researcher for Southern California Research; a consultant for Alladapt Immunotherapeutics Inc, Aluna, Hikma Pharmaceuticals, and Merck; an advisor for Amgen, AstraZeneca, and Hikma Pharmaceuticals; a speaker for AstraZeneca, Regeneron Pharmaceuticals, and Sanofi; and has an executive role/ownership interest in Allergy & Asthma Associates of Southern California. A. Assa’ad has received grant funding to their institution from AbbVie, Aimmune Therapeutics, Alladapt Immunotherapeutics Inc, DBV Technologies, FARE, 10.13039/100000002National Institutes of Health, Novartis, Sanofi, and Siolta; an internal research grant from Cincinnati Children’s Hospital Medical Centre; and is a holder of patent US7732135 (Genetic Marker of Food Allergy). S. Gogate has participated in advisory boards for IgGenix and Novartis. D. Petroni is a consultant for Aimmune Therapeutics, Alladapt Immunotherapeutics Inc, ARS Pharmaceuticals, DBV Technologies, Roche-Genentech, and Takeda Pharmaceuticals. T. Casale is a consultant for AstraZeneca, Genentech, Jasper Therapeutics, Lily, Novartis, Regeneron Pharmaceuticals, and Sanofi; and a member of the speakers’ bureau for Genentech and Novartis. O. Wang provided biostatistics support to Alladapt Immunotherapeutics Inc for the Harmony trial. M. Wang, A. Sullivan, A. Archer, C. Piscia-Nichols, L. Tuomi, O. Levin-Young, A. Dombkowski, and D. McClintock are current or previous employees of Alladapt Immunotherapeutics Inc.
