# Supplementary material for: ADP101 multifood oral immunotherapy for food-allergic patients: Harmony phase 1/2 randomized clinical trial
Source: J Allergy Clin Immunol Glob. 2024 Dec 10;4(1):100382. doi: 10.1016/j.jacig.2024.100382 (PMC11786640; doi:10.1016/j.jacig.2024.100382)
Supplement: Supplementary Figs and Tables [file mmc1.docx]

**Supplementary Appendix**

**For:**

**ADP101 Multifood Oral Immunotherapy for Food-Allergic Patients:
Harmony Phase 1/2 Randomized Clinical Trial**

Edwin H. Kim, MD, MS;^1^ Warner W. Carr, MD;^2^ Amal H. Assa’ad, MD;^3^ Shaila U. Gogate, MD;^4^ Daniel H. Petroni, MD, PhD;^5^ Thomas B. Casale, MD^6^; Mei-Lun Wang, MD;^7^ Amy Sullivan, BA; ^7^ Amy M. Archer, MD, PhD; ^7^ Ouhong Wang, PhD;^8^ Cheri Piscia-Nichols, BS; ^7^ Lisa Tuomi, PharmD;^7^ Olga Levin-Young, MBA;^7^ Ashley Dombkowski, PhD;^7^ and Dana McClintock, MD;^7^ on behalf of the Harmony investigators

^1^University of North Carolina School of Medicine, Department of Pediatrics, Chapel Hill, NC, USA; ^2^Allergy & Asthma Providers of Southern California, Mission Viejo, CA, USA; ^3^Cincinnati Children’s Hospital Medical Center, Cincinnati, OH, USA; ^4^ Colorado Allergy & Asthma Centers, P. C., Denver, CO, USA; ^5^Seattle Allergy and Asthma Research Institute, Seattle, WA, USA; ^6^University of South Florida, Tampa, FL, USA; ^7^Alladapt Immunotherapeutics, Inc., Menlo Park, CA, USA; ^8^PointOH5, LLC, Boston, MA, USA.

**Contents**

[Harmony Investigators 3](#_Toc175334010)

[Supplementary Methods 4](#_Toc175334011)

[Skin-prick test (SPT) 4](#_Toc175334012)

[Supplementary Results 4](#_Toc175334013)

[Exploratory analyses 4](#_Toc175334014)

[Results summary for adult participants ≥18 years of age 5](#_Toc175334015)

[Supplementary Figures 6](#_Toc175334016)

[Figure E1. CONSORT flow diagram for adult participants ≥18 years of age 6](#_Toc175334017)

[Figure E2. Change from baseline in SPT for qualifying reactive foods (pediatric ITT population) 7](#_Toc175334018)

[Figure E3. MTD for qualifying food allergies at Screening and Exit among participants who completed Exit DBPCFC (pediatric ITT population) 8](#_Toc175334019)

[Supplementary Tables 9](#_Toc175334020)

[Table E1. Harmony inclusion and exclusion criteria 9](#_Toc175334021)

[Table E2. DBPCFC up-dosing schedule 12](#_Toc175334022)

[Table E3. Screening and Exit DBPCFC results in adult participants ≥18 years of age 13](#_Toc175334023)

[Table E4. Nonqualifying reactive foods in the pediatric ITT population 14](#_Toc175334024)

[Table E5. Summary of treatment and exposure (pediatric participants) 15](#_Toc175334025)

[Table E6. Rates of tolerance to ≥600 mg of individual foods/food groups in Exit DBPCFC (qualifying food allergies; pediatric participants) 16](#_Toc175334026)

[Table E7. Summary of MTD, ED and fold-change from baseline by qualifying food allergy (pediatric ITT population) 18](#_Toc175334027)

[Table E8. Maximum severity of allergy symptoms at Screening and Exit DBPCFCs for qualifying foods (pediatric ITT population) 25](#_Toc175334028)

[Table E9. Summary of accidental exposures (pediatric ITT population) 27](#_Toc175334029)

[Table E10. Day 1 TEAEs, and TEAEs occurring in ≥2 participants in any treatment group by treatment phase (pediatric safety population) 28](#_Toc175334030)

[Table E11. TEAEs reported by ≥20% of participants in any treatment group (pediatric population) 30](#_Toc175334031)

[Table E12. TEAEs related to study drug in ≥2 participants in any treatment group, by treatment period (pediatric population) 31](#_Toc175334032)

[Table E13. Food equivalents in milligrams of protein 32](#_Toc175334033)

[References 34](#_Toc175334034)

# Harmony Investigators

| **Investigator** | **Affiliation*** | **IRB/Ethics committee** |
| --- | --- | --- |
| Sunena C. Argo, MD | Clinical Research Center of Alabama, Birmingham, AL | Advarra IRB, Columbia, MD |
| Amal H. Assa'ad, MD | Cincinnati Children's Hospital Medical Center, Cincinnati, OH | Cincinnati Children's Hospital IRB, Cincinnati, OH |
| Terri Brown-Whitehorn, MD | Children's Hospital of Philadelphia, Philadelphia, PA | The Children's Hospital of Philadelphia IRB, Philadelphia, PA |
| Warner W. Carr, MD | Allergy & Asthma Associates of Southern California, Mission Viejo, CA | Advarra IRB, Columbia, MD |
| Thomas B. Casale, MD | University of South Florida, Tampa, FL | University of South Florida IRB, Tampa, FL (initial submission); Advarra IRB, Columbia, MD (subsequent submissions) |
| Stanley M. Fineman, MD, MBA | Atlanta Allergy & Asthma Clinic, Marietta, GA | Advarra IRB, Columbia, MD |
| Stephen B. Fritz, MD | Portland Allergy and Asthma, Happy Valley, OR | Advarra IRB, Columbia, MD |
| Shaila U. Gogate, MD | Colorado Allergy and Asthma Centers, Denver, CO | Advarra IRB, Columbia, MD |
| Alexander N. Greiner, MD | Allergy & Asthma Medical Group and Research Center, San Diego, CA | Advarra IRB, Columbia, MD |
| Edwin H. Kim, MD, MS | UNC Chapel Hill, Chapel Hill, NC | Advarra IRB, Columbia, MD |
| Anna Nowak-Wegrzyn, MD, PhD | NYU Langone Health, New York, NY | NYU Grossman School of Medicine IRB, New York, NY |
| Daniel H. Petroni, MD, PhD | Seattle Allergy and Asthma Research Institute, Seattle, WA | Advarra IRB, Columbia, MD |
| Ned T. Rupp, MD | National Allergy and Asthma Research, N. Charleston, SC | Advarra IRB, Columbia, MD |
| Georgiana M. Sanders, MD | University of Michigan, Ann Arbor, MI | University of Michigan IRB, Ann Arbor, MI (initial submission); Advarra IRB, Columbia, MD (subsequent submissions) |
| Lawrence D. Sher, MD | Peninsula Research Associates, Rolling Hills Estates, CA | Advarra IRB, Columbia, MD |
| Dareen Siri, MD | Sneeze, Wheeze, & Itch Associates, Normal, IL | Advarra IRB, Columbia, MD |
| Brian P. Vickery, MD | Children's Healthcare of Atlanta, Atlanta, GA | Advarra IRB, Columbia, MD |

*17 study centers were activated in the United States; 15 enrolled participants in the trial.

# Supplementary Methods

## Skin-prick test (SPT)

SPT results were obtained during the Screening period for each of the 15 foods in ADP101, to identify foods to be further evaluated in double-blind, placebo-controlled food challenge (DBPCFC) at Screening. To assess change from baseline, participants underwent SPT for all 15 foods again at Week 38. Week 38 SPT results were also used to determine whether foods deemed nonreactive at baseline would be tested by DBPCFC at Week 40.

# Supplementary Results

## Exploratory analyses

*Skin-prick test (SPT)*Decreases from baseline in mean wheal diameter (relative to negative control) were observed at Week 38 in the HD-ADP101 group for hazelnut and walnut, and in both the LD-ADP101 and HD-ADP101 group for cashew, chicken’s egg, peanut and pistachio. Interpretation of results for other foods was limited due to the small sample size. Median and individual SPT values across all food types are shown in Figure E2, with individual values separated by responder/nonresponder, and whether or not the target dose was reached. For ADP101-treated groups, median SPT values showed a downward trend at Week 38, while the pooled placebo group showed an upward trend.

*Maximum tolerated dose (MTD) and eliciting dose (ED)*
The MTD over time for qualifying food allergies by participant and food is shown in Figure E3, with a summary of MTD and ED by qualifying food shown in Table E7. In the pediatric ITT population, comparisons between placebo and ADP101 could be made for 9 individual foods; almond, cashew, chicken’s egg, cow’s milk, hazelnut, peanut, pecan, pistachio, and walnut; with at least 1 participant in the placebo group and at least 1 in either ADP101 group. The median change from baseline in MTD and ED, as well as corresponding fold-changes, were numerically greater in either or both ADP101 treatment groups compared with placebo in all of these foods with the exception of hazelnut.

There were no participants in the placebo group with qualifying allergy to cod, salmon, sesame, shrimp and wheat, precluding any comparison between ADP101 and placebo. However, MTD and ED were found to increase between Screening and Exit DBPCFC in all pediatric ADP101-treated participants who completed Exit DBPCFC for these foods. Numerically greater differences between HD-ADP101 and placebo were observed in ED fold-change between Screening and Exit DBPCFC for cashew (nominal *P* = 0.017), peanut (nominal *P* = 0.004), and pistachio (nominal *P* = 0.042). There were no pediatric participants with a qualifying soy allergy.

*Maximum severity of allergy symptoms at Screening and Exit DBPCFC*
Maximum severity of allergy symptoms by qualifying food is summarized in Table E8. The incidence of severe symptoms was rare, occurring in 4 participants in the pediatric ITT population: 2 during Screening DBPCFC (LD-ADP101 group, sesame seed; and HD-ADP101 group, cashew), and 2 during Exit DBPCFC (placebo group, cow’s milk; and HD-ADP101 group, chicken’s egg). Comparison between placebo and ADP101 treatment groups could be made across 9 individual foods; almond, cashew, chicken’s egg, cow’s milk, hazelnut, peanut, pecan, pistachio and walnut. Generally, the reduction in maximum severity of symptoms from Screening to Exit DBPCFC was greater in ADP101-treated participants than with placebo.

*Neosensitization*The development of new food allergies (neosensitization) was evaluated at the Exit DBPCFC in participants with new clinical symptoms and/or a threshold increase in food-specific SPT from baseline. Dose-limiting symptoms in response to a previously nonreactive food were considered to be neosensitization events. One participant in the pooled placebo group and 1 participant in the LD-ADP101 group met the definition for neosensitization; both cases occurred with hazelnut, in participants with other confirmed tree nut allergies at baseline.

*Accidental exposure*
Accidental exposure occurred in 18.0% (11/61) of participants; in 4, 2, and 5 participants in the placebo, LD-ADP101, and HD-ADP101 groups, respectively (Table E9). Seven participants required treatment as a result of accidental exposure; 2 received epinephrine for Grade 2 anaphylaxis (1 in the placebo group, 1 HD-ADP101). All AEs due to accidental exposure were mild to moderate in severity, and none required hospitalization.

## Results summary for adult participants ≥18 years of age

Harmony enrolled 12 adult participants (≥18 years of age) as a feasibility cohort for exploratory assessment of ADP101 efficacy and safety in this population (Figure E1). Adult participants ranged in age from 18 to 46 years (median, 32.5 years), were 75.0% (9/12) female and 91.7% (11/12) white; 1 participant was Asian. Treatment and Exit DBPCFC were completed by 100.0% (4/4) of pooled placebo, 100.0% (4/4) of LD-ADP101, and 25.0% (1/4) of HD-ADP101 participants. Three participants, all in the HD-ADP101 treatment group, discontinued for reasons of adverse events (n=2) and withdrawal by participant (n=1).

Reactive food allergies at Screening and Exit DBPCFC are listed in Table E3. Toleration of ≥1 qualifying food at the ≥600 mg challenge dose level without dose-limiting symptoms was achieved by 2/4 pooled placebo, 2/4 LD-ADP101, and 0/4 HD-ADP101-treated participants (3 did not complete the Exit DBPCFC). There were no cases of neosensitization among adult participants.

Treatment-emergent adverse events (TEAEs) occurred in all 12 adult participants, with study-drug related TEAEs occurring in 100.0% (4/4) of pooled placebo, 75.0% (3/4) of LD-ADP101, and 100% (4/4) of HD-ADP101 participants. TEAEs leading to trial discontinuation occurred in 2 participants, both in the HD-ADP101 group (1 participant with Grade 3 anaphylactic reaction, 1 with Grade 1 vomiting). One further participant in the HD-ADP101 group experienced Grade 3 anaphylaxis but remained on-treatment with dose reduction.

There were no serious adverse events (SAEs) or life-threatening (Grade 4) TEAEs reported in the adult population, and no deaths.

# **Supplementary Figures**

## Figure E1. CONSORT flow diagram for adult participants ≥18 years of age

*DBPCFC, double-blind, placebo-controlled food challenge; OLE, open-label extension.*

**
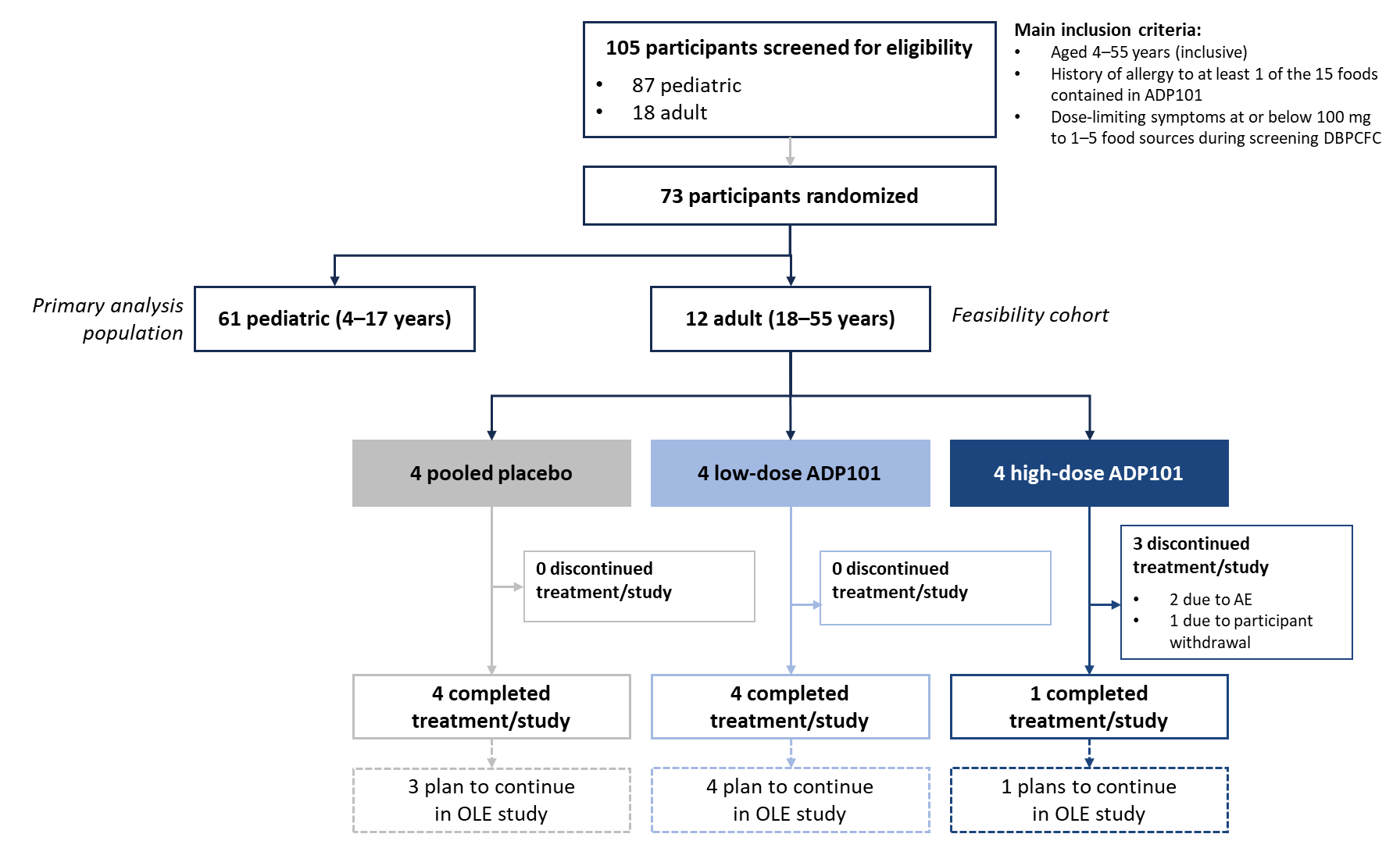
**

## **Figure E2. Change from baseline in SPT for qualifying reactive foods (pediatric ITT population)**

Exploratory endpoint. *One response not shown: a wheal diameter of 54 mm at baseline (relative to negative control) with no corresponding Week 38 assessment.
*IQR, inter-quartile range; HD, high-dose; LD, low-dose; SPT, skin-prick test.*

*
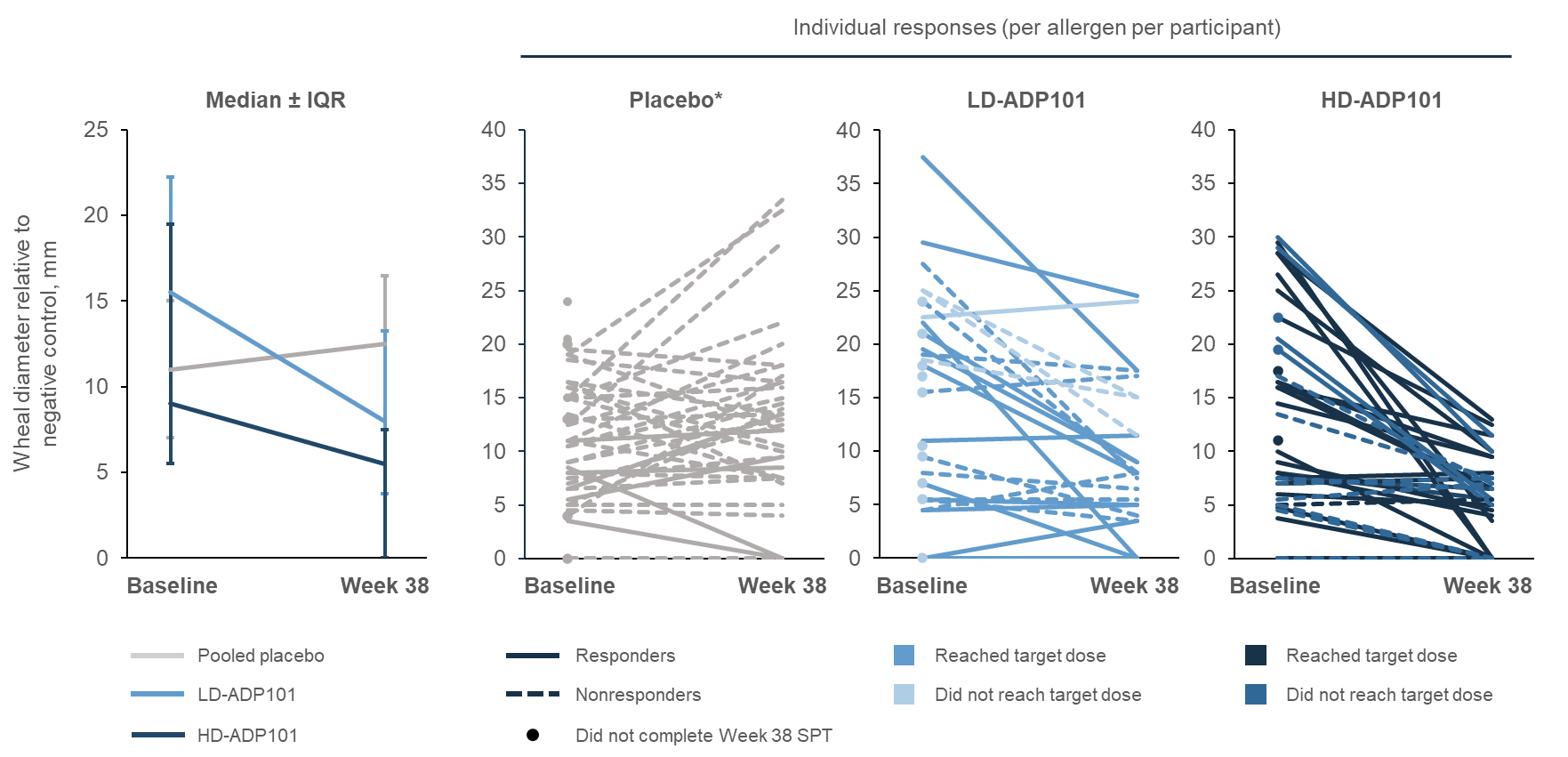
*

## Figure E3. MTD for qualifying food allergies at Screening and Exit among participants who completed Exit DBPCFC (pediatric ITT population)

Exploratory endpoint. *DBPCFC, double-blind, placebo-controlled food challenge; HD, high-dose; LD, low-dose; MTD, maximum tolerated dose.*

**
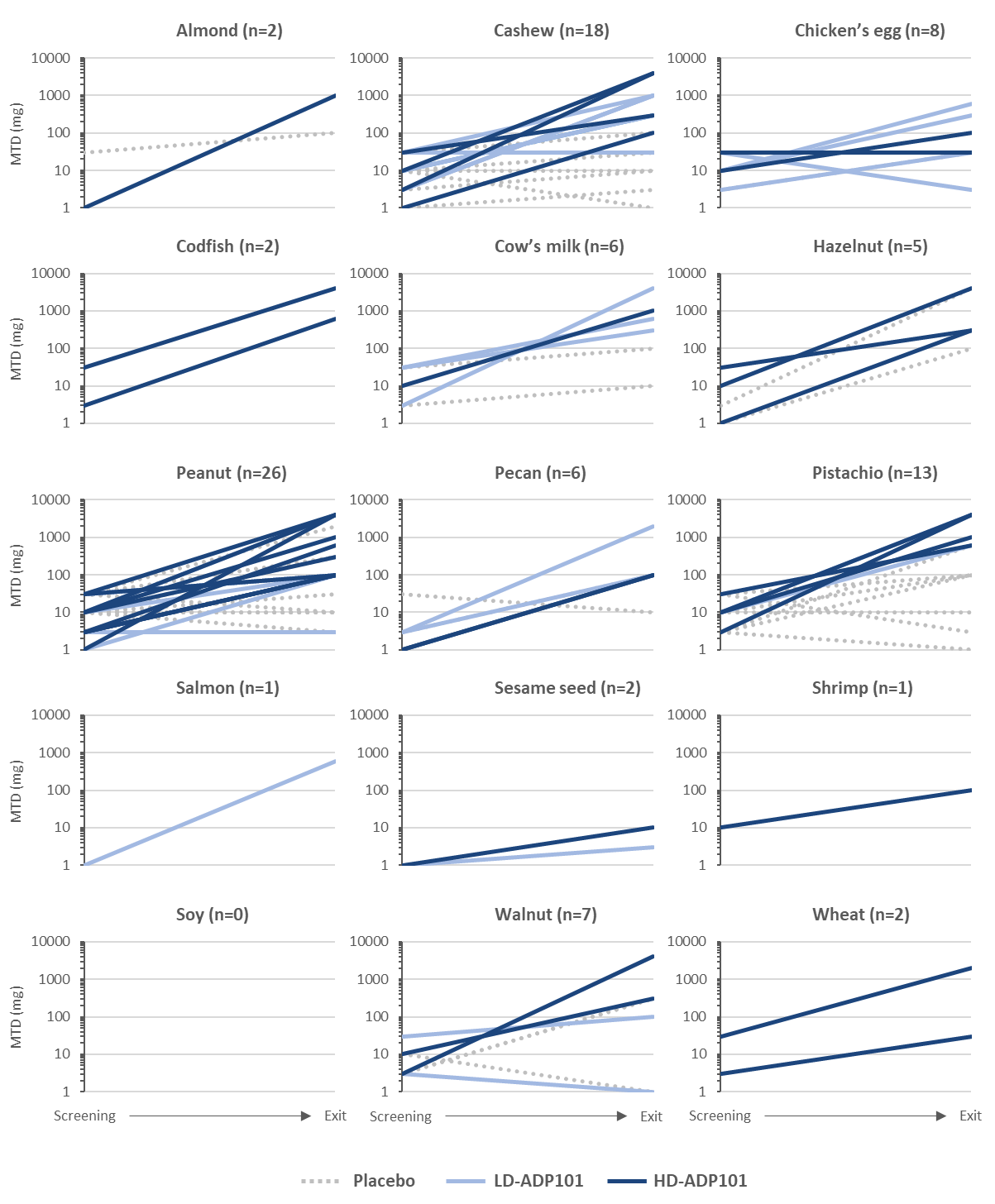
**

# Supplementary Tables

## Table E1. Harmony inclusion and exclusion criteria

| **Inclusion Criteria** | For inclusion in the trial, participants were required to fulfill all of the following criteria: |
| --- | --- |
| **Participant and Disease Characteristics** | - Aged 4 to 55 years (inclusive) at the time of signing the ICF - Clinical history of allergy to ≥1 of the foods contained in ADP101 (i.e., almond, cashew, chicken’s egg, codfish, cow’s milk, hazelnut, peanut, pecan, pistachio, salmon, sesame seed, shrimp, soy, walnut, and wheat) - Experience dose-limiting symptoms at or below the 100-mg dose level to ≥1 food source and ≤5 food sources during the Screening DBPCFC |
| **Contraception** | - All participants who were of childbearing potential and their partners must have agreed to use highly effective contraception during the trial. All participants and their partners must have continued to use highly effective contraception for 30 days after the last dose of study drug. - Female participants of childbearing potential must have had a negative serum pregnancy test at Screening and a negative urine pregnancy test at baseline. - Female participants not of childbearing potential must have been either premenstrual or postmenopausal (defined as cessation of regular menstrual periods for ≥12 months) and were not required to use contraception. |
| **Informed Consent** | - Participant and/or legally authorized representative (i.e., parent/guardian) must have been capable of giving signed informed consent, which included compliance with the requirements and restrictions listed on the ICF. - Minor participants must have been capable of giving written assent as appropriate per the applicable age (per local regulatory requirements). |
| **Exclusion Criteria** | Any of the following was regarded as a criterion for exclusion from the trial: |
| **Medical Conditions** | - Confirmed presence of >5 FAs with dose-limiting symptoms at or below the 100-mg challenge dose level during the Screening DBPCFC of food sources contained in ADP101 - History of severe or life-threatening episode(s) of anaphylaxis or anaphylactic shock within 60 days of the first Screening DBPCFC - History of EoE; other eosinophilic gastrointestinal disease; chronic, recurrent, or severe gastrointestinal reflux disease (GERD); symptoms of dysphagia (e.g., difficulty swallowing, food “getting stuck”); or recurrent gastrointestinal symptoms of undiagnosed etiology - History of chronic disease (other than asthma, atopic dermatitis, or allergic rhinitis) that was, or was at significant risk of becoming, unstable or requiring a change in chronic therapeutic regimen - Severe asthma per 2007 National Heart, Lung, and Blood Institute (NHLBI) Criteria Steps 5 or 6 - Mild or moderate asthma (2007 NHLBI Criteria Steps 1 through 4), if uncontrolled or difficult to control, as defined by any of the following:   - FEV_1_ <80% of predicted, or ratio of FEV_1_ to forced vital capacity (FVC) <75% of predicted, with or without controller medications (only for age 6 years or greater and able to do spirometry), *OR*   - Inhaled corticosteroid (ICS) dosing of >500 μg daily fluticasone (or equivalent ICS based on NHLBI dosing chart), *OR*   - One hospitalization in the previous year for asthma prior to Screening, *OR*   - Emergency room visit for asthma within 6 months prior to Screening - Known malignancy that was progressing or required active treatment within the past 3 years. *Note:* Participants with basal cell carcinoma of the skin, squamous cell carcinoma of the skin, or carcinoma in situ (e.g., breast carcinoma, cervical cancer in situ) that had undergone potentially curative therapy were not excluded. - Known history of human immunodeficiency virus (HIV) (HIV 1/2 antibodies). *Note:* No testing for HIV was required unless mandated by the local health authority. - Known active hepatitis B infection (defined as hepatitis B surface antigen   [HBsAg]-reactive) or known active hepatitis C virus (HCV) infection (defined as HCV RNA [qualitative] detected). *Note:* No testing for hepatitis B and hepatitis C was required unless mandated by the local health authority.   - Uncontrolled diabetes. *Note:* Participants with controlled diabetes were allowed (insulin was allowed). - Hypertension requiring >2 antihypertensive medications - Known history of cardiovascular disease, including, but not limited to, history of myocardial infarction or arterial thromboembolic events within 6 months prior to enrollment, severe or unstable angina, New York Heart Association (NYHA) Class III or IV disease, or a history of corrected QT (QTc) interval >470 ms. ^1^ *Note:* Well-controlled hypertension was not considered to be part of this definition; see above - History of interstitial lung disease - History of confirmed mast cell disorder, including mastocytosis, urticaria pigmentosa, or hereditary or idiopathic angioedema - History of celiac disease and/or any significant non-IgE–mediated intolerance (e.g., severe lactose intolerance) to ≥1 of the food sources contained in ADP101 - Active infection within 30 days of Screening requiring systemic therapy - Female participants who were pregnant or breastfeeding, or were expecting to conceive, or male participants planning to father children within the projected duration of the trial - Active autoimmune disease that required systemic treatment within 3 months of trial entry (i.e., use of disease-modifying agents, corticosteroids, or immunosuppressive drugs) - Known psychiatric or substance-abuse disorders that would interfere with cooperation with the requirements of the trial - History or current evidence of any condition, therapy, or clinically significant laboratory abnormality that might have precluded safe participation, confounded the results of the trial, or interfered with the participant’s participation for the full duration of the trial, or for which it was not in the best interest of the participant to participate, in the opinion of the treating investigator |
| **Prior/Concurrent Therapy (Relative to Screening)** | - History of regular steroid medication use (via intravenous, intramuscular, or oral administration) in any of the following manners:   - History of daily oral steroid dosing for >1 month during the previous year, *OR*   - Burst oral steroid course in the previous 1 month, *OR*   - >2 burst oral steroid courses in the previous year ≥1 week in duration - In the “build-up phase” of immunotherapy (i.e., had not reached maintenance dosing for ≥2 weeks) to another non-food allergen (e.g., environmental allergen, bee venom, etc.) - Inability to discontinue antihistamines ≥5 half-lives before SPT and DBPCFC - Use of any therapeutic antibody (e.g., dupilumab, omalizumab, mepolizumab, reslizumab) currently or within 6 months or 5 half-lives prior to trial entry, whichever was longer - Use of any food immunotherapy (e.g., oral, sublingual, epicutaneous) currently or within the 12 weeks prior to trial entry - Use of beta-blockers (oral), angiotensin-converting enzyme (ACE) inhibitors,   angiotensin-receptor blockers (ARBs), or calcium-channel blockers |
| **Prior/Concurrent Clinical Trial Experience** | - Current participation or had participated in a trial of an investigational agent within 4 weeks prior to Screening or 5 half-lives of the other investigational agent, whichever was longer |
| **Other Exclusions** | - Developed dose-limiting symptoms to placebo during the Screening DBPCFC - Hypersensitivity to epinephrine or any of the excipients in trial drug - Resided at the same address as another participant (e.g., siblings) participating in this or any other OIT/mOIT trial |

*DBPCFC, double-blind placebo-controlled food challenge; EoE, eosinophilic esophagitis; FA, food allergy; FEV_1_, forced expiratory volume in the first second; ICF, informed consent form; mOIT multifood oral immunotherapy; OIT, oral immunotherapy; SPT, skin-prick test.*

## Table E2. DBPCFC up-dosing schedule

| **Type of Test** | **Screening** | **Exit** |
| --- | --- | --- |
| **Dose (cumulative total dose), mg** | 1 | 1 |
|  | 3 (4) | 3 (4) |
|  | 10 (14) | 10 (14) |
|  | 30 (44) | 30 (44) |
|  | 100 (144) | 100 (144) |
|  | 300 (444) | 300 (444) |
|  | 600 (1044) | 600 (1044) |
|  | 1000 (2044) | 1000 (2044) |
|  |  | 2000 (4044) |
|  |  | 4000 (8044) |
| **Comments** | The Screening DBPCFC consisted of 8 doses of food source given every 15–30 minutes in increasing amounts up to 1000 mg (2044 mg cumulative) of food protein (1 hour maximum between doses). | The Exit DBPCFC consisted of 10 doses of food source given every 15–30 minutes in increasing amounts up to 4000 mg (8044 mg cumulative) of food protein (1 hour maximum between doses). |

*DBPCFC, double-blind, placebo-controlled food challenge.*

## Table E3. Screening and Exit DBPCFC results in adult participants ≥18 years of age

| **Participant** | **Treatment Group** | **Reactive Foods at Screening** | **Screening**  **MTD, eliciting dose (mg)** | **Exit DBPCFC**  **MTD, eliciting dose (mg)** |
| --- | --- | --- | --- | --- |
| **1** | **LD-placebo** | Peanut | 10, 30 | 100, 300 |
| **2** | **LD-placebo** | Cashew | 30, 100 | **4000, 4000** |
|  |  | Hazelnut | 600, 1000 *(Non-QFA)* | 100, 300 |
|  |  | Peanut | 10, 30 | 100, 300 |
|  |  | Pistachio | 10, 30 | **4000, 4000** |
| **3** | **HD-placebo** | Shrimp | 3, 10 | 300, 600 |
| **4** | **HD-placebo** | Cashew | 10, 30 | **600, 1000** |
|  |  | Pistachio | 30, 100 | **600, 1000** |
|  |  | Salmon | 3, 10 | 100, 300 |
|  |  | Wheat | 30, 100 | **4000, 4000** |
| **5** | **LD-ADP101** | Cashew | 3, 10 | 100, 300 |
|  |  | Pistachio | 30, 100 | **4000, 4000** |
| **6** | **LD-ADP101** | Peanut | 10, 30 | 300, 600 |
| **7** | **LD-ADP101** | Peanut | 1, 1 | 300, 600 |
| **8** | **LD-ADP101** | Hazelnut | 1, 3 | **4000, 4000** |
|  |  | Shrimp | 3, 10 | **4000, 4000** |
|  |  | Soy | 10, 30 | 300, 600 |
| **9** | **HD-ADP101** | Peanut | 3, 10 | 100, 300 |
| **10** | **HD-ADP101** | Peanut | 3, 10 | *Did not complete* |
|  |  | Soy | 300, 600 *(Non-QFA)* |  |
| **11** | **HD-ADP101** | Chicken’s egg | 3, 10 | *Did not complete* |
|  |  | Peanut | 30, 100 |  |
| **12** | **HD-ADP101** | Sesame | 1, 1 | *Did not complete* |

Bold indicates responders at the ≥600-mg dose level. If ED was the lowest dose of 1 mg, MTD was also considered to be 1 mg.
*DBPCFC, double-blind, placebo-controlled food challenge; HD, high-dose; LD, low-dose; MTD, maximum tolerated dose; QFA, qualifying food allergy.*

## **Table E4. Nonqualifying reactive foods in the pediatric ITT population**

| **Characteristic** | **Pooled placebo (N=20)** | **LD ADP101**  **(N=21)** | **HD ADP101 (N=20)** |
| --- | --- | --- | --- |
| **Nonqualifying* food allergies per participant based on Screening DBPCFC, n (%)** |  |  |  |
| 1 | 10 (50.0) | 5 (23.8) | 6 (30.0) |
| 2 | 0 | 3 (14.3) | 2 (10.0) |
| 3 | 1 (5.0) | 1 (4.8) | 0 |
| 4 | 0 | 0 | 0 |
| ≥5 | 0 | 0 | 0 |
| **Individual nonqualifying food allergies based on Screening DBPCFC, n (%)** |  |  |  |
| Pistachio | 4 (20.0) | 3 (14.3) | 2 (10.0) |
| Peanut | 3 (15.0) | 3 (14.3) | 0 |
| Pecan | 2 (10.0) | 1 (4.8) | 1 (5.0) |
| Sesame Seed | 1 (5.0) | 1 (4.8) | 2 (10.0) |
| Walnut | 1 (5.0) | 2 (9.5) | 1 (5.0) |
| Cashew | 1 (5.0) | 0 | 2 (10.0) |
| Chicken’s egg | 0 | 1 (4.8) | 1 (5.0) |
| Codfish | 1 (5.0) | 1 (4.8) | 0 |
| Cow’s milk | 0 | 0 | 1 (5.0) |
| Hazelnut | 0 | 1 (4.8) | 0 |
| Shrimp | 0 | 1 (4.8) | 0 |
| Almond | 0 | 0 | 0 |
| Salmon | 0 | 0 | 0 |
| Soy | 0 | 0 | 0 |
| Wheat | 0 | 0 | 0 |

*Nonqualifying reactive food defined as a food that elicited a reaction at >100 mg but ≤1000 mg during Screening DBPCFC.
*DBPCFC, double-blind, placebo-controlled food challenge; HD, high-dose; LD, low-dose; N, number of participants in the specified analysis population under each group; n, number of participants in the specified group.*

## **Table E5. Summary of treatment and exposure (pediatric participants**)

| **Category** | **Pooled placebo (N=20)** | **LD ADP101**  **(N=21)** | **HD ADP101 (N=20)** |
| --- | --- | --- | --- |
| Mean treatment compliance, % (SD) | 88.4 (14.2) | 91.0 (13.9) | 93.0 (8.6) |
| Participants with ≥90% treatment compliance, n (%) | 14 (70.0) | 17 (81.0) | 15 (75.0) |
| Mean number of missed doses, n (SD) | 36.5 (43.4) | 26.7 (48.5) | 23.2 (29.5) |
| Median overall exposure to study drug, days | 333.5 | 321.0 | 331.0 |
| Participants achieving target dose level, n (%) | 18 (90.0) | 16 (76.2) | 14 (70.0) |
| Median time to reach target dose level, weeks (95% CI) | 20.6 (19.1–22.7) | 20.6 (17.1–28.3) | 24.2 (20.1–28.0) |
| Participants achieving target dose for maintenance, n (%) | 18 (90.0) | 14 (66.7) | 13 (65.0) |
| Median time at maintenance dose, days (range) | 142.0 (27–175) | 145.0 (14–207) | 112.0 (17–150) |

*CI, confidence interval; HD, high-dose; LD, low-dose; N, number of participants in the specified analysis population under each group; n, number of participants in the specified group; SD, standard deviation.*

## **Table E6. Rates of tolerance to ≥600 mg of individual foods/food groups in Exit DBPCFC (qualifying food allergies; pediatric participants)**

| **Qualifying food/food group** | **Pooled placebo (N=20)** | **LD ADP101**  **(N=21)** | **HD ADP101 (N=20)** |
| --- | --- | --- | --- |
| **Peanut** |  |  |  |
| Participants with QFA (N1) | 11 | 9 | 12 |
| Participants with ≥600 mg desensitization response (n) | 1 | 1 | 5 |
| Proportion (95% CI)* | 0.091 (0.002, 0.413) | 0.111 (0.003, 0.482) | 0.417 (0.152, 0.723) |
| Difference (ADP101 minus placebo) |  | 0.020 | 0.326 |
| Unadjusted *P*-value† |  | >0.999 | 0.155 |
| **Tree nut** |  |  |  |
| Participants with QFA (N1) | 14 | 9 | 7 |
| Participants with ≥600 mg desensitization response (n) | 3 | 4 | 5 |
| Proportion (95% CI)* | 0.214 (0.047, 0.508) | 0.444 (0.137, 0.788) | 0.714 (0.290, 0.963) |
| Difference (ADP101 minus placebo) |  | 0.230 | 0.500 |
| Unadjusted *P*-value† |  | 0.363 | 0.056 |
| **Chicken’s egg** |  |  |  |
| Participants with QFA (N1) | 1 | 5 | 3 |
| Participants with ≥600 mg desensitization response (n) | 0 | 1 | 0 |
| Proportion (95% CI)* | 0 (0.000, 0.975) | 0.200 (0.005, 0.716) | 0 (0.000, 0.708) |
| Difference (ADP101 minus placebo) |  | 0.200 | NA |
| Unadjusted *P*-value† |  | >0.999 | NA |
| **Cow’s milk** |  |  |  |
| Participants with QFA (N1) | 3 | 3 | 1 |
| Participants with ≥600 mg desensitization response (n) | 0 | 2 | 1 |
| Proportion (95% CI)* | 0 (0.000, 0.708) | 0.667 (0.094, 0.992) | 1.000 (0.025, 1.000) |
| Difference (ADP101 minus placebo) |  | 0.667 | 1.000 |
| Unadjusted *P*-value† |  | 0.400 | 0.250 |
| **Finfish** |  |  |  |
| Participants with QFA (N1) | 0 | 1 | 2 |
| Participants with ≥600 mg desensitization response (n) | 0 | 1 | 2 |
| Proportion (95% CI)* | NA | 1.000 (0.025, 1.000) | 1.000 (0.158, 1.000) |
| Difference (ADP101 minus placebo) |  |  |  |
| Unadjusted *P*-value† |  |  |  |
| **Sesame seed** |  |  |  |
| Participants with QFA (N1) | 0 | 2 | 1 |
| Participants with ≥600 mg desensitization response (n) | 0 | 0 | 0 |
| Proportion (95% CI)* | NA | 0 (0.000, 0.842) | 0 (0.000, 0.975) |
| Difference (ADP101 minus placebo) |  |  |  |
| Unadjusted *P*-value† |  |  |  |
| **Shrimp** |  |  |  |
| Participants with QFA (N1) | 0 | 0 | 2 |
| Participants with ≥600 mg desensitization response (n) | 0 | 0 | 0 |
| Proportion (95% CI)* | NA | NA | 0 (0.000, 0.842) |
| Difference (ADP101 minus placebo) |  |  |  |
| Unadjusted *P*-value† |  |  |  |
| **Wheat** |  |  |  |
| Participants with QFA (N1) | 0 | 0 | 2 |
| Participants with ≥600 mg desensitization response (n) | 0 | 0 | 1 |
| Proportion (95% CI)* | NA | NA | 0.500 (0.013, 0.987) |
| Difference (ADP101 minus placebo) |  |  |  |
| Unadjusted *P*-value† |  |  |  |
| **Soy** |  |  |  |
| Participants with QFA (N1) | 0 | 0 | 0 |
| Participants with ≥600 mg desensitization response (n) | 0 | 0 | 0 |
| Proportion (95% CI)* | NA | NA | NA |
| Difference (ADP101 minus placebo) |  |  |  |
| Unadjusted *P*-value^†^ |  |  |  |

Prespecified subgroup analysis. Desensitization response defined as tolerating ≥600 mg of food protein without dose-limiting symptoms on Exit DBPCFC, for at least 1 qualifying food in the category. Finfish includes codfish and salmon; tree nut includes almond, cashew, hazelnut, pecan, pistachio, and walnut.
*95% CI of proportion estimated using Clopper-Pearson exact method. †Unadjusted *P*-value determined using Fisher’s exact test.
*DBPCFC, double-blind, placebo-controlled food challenge; HD, high-dose; LD, low-dose; N, number of participants in the specified analysis population under each treatment group; n, number of responders; N1, number of participants for each qualifying food/food group; NA, not applicable; QFA, qualifying food allergy.*

## Table E7. Summary of MTD, ED and fold-change from baseline by qualifying food allergy (pediatric ITT population)

| **Category, n (%)** | **Pooled placebo (N=20)** | **LD ADP101**  **(N=21)** | **HD ADP101 (N=20)** |
| --- | --- | --- | --- |
| **Almond** |  |  |  |
| Participants with QFA, n | 1 | 0 | 1 |
| MTD |  |  |  |
| Screening DBPCFC, n | 1 | 0 | 1 |
| Median, mg (range) | 30.0 (30.0–30.0) |  | 1.0 (1.0–1.0) |
| Exit DBPCFC, n | 1 | 0 | 1 |
| Median, mg (range) | 100.0 (100.0–100.0) |  | 1000.0 (1000.0-1000.0) |
| Fold change from Screening DBPCFC, n | 1 | 0 | 1 |
| Median (min/max) | 3.33 (3.3–3.3) |  | 1000.0 (1000.0–1000.0) |
| Eliciting dose |  |  |  |
| Screening DBPCFC, n | 1 | 0 | 1 |
| Median, mg (range) | 100.0 (100.0–100.0) |  | 3.0 (3.0–3.0) |
| Exit DBPCFC, n | 1 | 0 | 1 |
| Median, mg (range) | 300.0 (300.0–300.0) |  | 2000.0 (2000.0–2000.0) |
| *P*-value (Exit versus Screening DBPCFC)* | >0.999 | NA | >0.999 |
| Fold change from Screening DBPCFC, n | 1 | 0 | 1 |
| Median (min/max) | 3.0 (3.0–3.0) |  | 666.7 (666.7–666.7) |
| *P*-value (ADP101 versus placebo)† |  | NA | NA |
| **Cashew** |  |  |  |
| Participants with QFA, n | 10 | 7 | 5 |
| MTD |  |  |  |
| Screening DBPCFC, n | 10 | 7 | 5 |
| Median, mg (range) | 10.0 (1.0–30.0) | 10.0 (1.0–30.0) | 10.0 (1.0–30.0) |
| Exit DBPCFC, n | 9 | 5 | 4 |
| Median, mg (range) | 30.0 (1.0–1000.0) | 1000.0 (30.0–1000.0) | 2150.0 (100.0-4000.0) |
| Fold change from Screening DBPCFC, n | 9 | 5 | 4 |
| Median (min/max) | 3.0 (0.1–333.3) | 33.3 (1.0–333.3) | 250.0 (10.0–1333.3) |
| Eliciting dose |  |  |  |
| Screening DBPCFC, n | 10 | 7 | 5 |
| Median, mg (range) | 30.0 (3.0–100.0) | 30.0 (3.0–100.0) | 30.0 (1.0–100.0) |
| Exit DBPCFC, n | 9 | 5 | 4 |
| Median, mg (range) | 100.0 (1.0–2000.0) | 2000.0 (100.0–2000.0) | 2300.0 (300.0–4000.0) |
| *P*-value (Exit versus Screening DBPCFC)* | 0.078 | 0.125 | 0.125 |
| Fold change from Screening DBPCFC, n | 9 | 5 | 4 |
| Median (min/max) | 3.0 (0.0–200.0) | 20.0 (1.0–200.0) | 216.7 (6.0–400.0) |
| *P*-value (ADP101 versus placebo)† |  | 0.138 | 0.017 |
| **Chicken’s Egg** |  |  |  |
| Participants with QFA, n | 1 | 5 | 3 |
| MTD |  |  |  |
| Screening DBPCFC, n | 1 | 5 | 3 |
| Median, mg (range) | 3.0 (3.0–3.0) | 10.0 (3.0–30.0) | 30.0 (10.0–30.0) |
| Exit DBPCFC, n | 1 | 4 | 3 |
| Median, mg (range) | 30.0 (30.0–30.0) | 165.0 (3.0–600.0) | 30.0 (30.0-100.0) |
| Fold change from Screening DBPCFC, n | 1 | 4 | 3 |
| Median (min/max) | 10.0 (10.0–10.0) | 20.0 (0.1–60.0) | 1.0 (1.0–10.0) |
| Eliciting dose |  |  |  |
| Screening DBPCFC, n | 1 | 5 | 3 |
| Median, mg (range) | 10.0 (10.0–10.0) | 30.0 (10.0–100.0) | 100.0 (30.0–100.0) |
| Exit DBPCFC, n | 1 | 4 | 3 |
| Median, mg (range) | 100.0 (100.0–100.0) | 350.0 (10.0–1000.0) | 100.0 (100.0–300.0) |
| *P*-value (Exit versus Screening DBPCFC)* | >0.999 | 0.375 | >0.999 |
| Fold change from Screening DBPCFC, n | 1 | 4 | 3 |
| Median (min/max) | 10.0 (10.0–10.0) | 15.0 (0.1–33.3) | 1.0 (1.0–10.0) |
| *P*-value (ADP101 versus placebo)† |  | >0.999 | >0.999 |
| **Codfish** |  |  |  |
| Participants with QFA, n | 0 | 0 | 2 |
| MTD |  |  |  |
| Screening DBPCFC, n | 0 | 0 | 2 |
| Median, mg (range) |  |  | 16.5 (3.0–30.0) |
| Exit DBPCFC, n | 0 | 0 | 2 |
| Median, mg (range) |  |  | 2300.0 (600.0–4000.0) |
| Fold change from Screening DBPCFC, n | 0 | 0 | 2 |
| Median (min/max) |  |  | 166.7 (133.3–200.0) |
| Eliciting dose |  |  |  |
| Screening DBPCFC, n | 0 | 0 | 2 |
| Median, mg (range) |  |  | 55.0 (10.0–100.0) |
| Exit DBPCFC, n | 0 | 0 | 2 |
| Median, mg (range) |  |  | 2500.0 (1000.0–4000.0) |
| *P*-value (Exit versus Screening DBPCFC)* | NA | NA | 0.5 |
| Fold change from Screening DBPCFC, n | 0 | 0 | 2 |
| Median (min/max) |  |  | 70.0 (40.0–100.0) |
| *P*-value (ADP101 versus placebo)† |  | NA | NA |
| **Cow’s milk** |  |  |  |
| Participants with QFA, n | 3 | 3 | 1 |
| MTD |  |  |  |
| Screening DBPCFC, n | 3 | 3 | 1 |
| Median, mg (range) | 30.0 (3.0–30.0) | 30.0 (3.0–30.0) | 10.0 (10.0–10.0) |
| Exit DBPCFC, n | 2 | 3 | 1 |
| Median, mg (range) | 55.0 (10.0–100.0) | 600.0 (300.0–4000.0) | 1000.0 (1000.0-1000.0) |
| Fold change from Screening DBPCFC, n | 2 | 3 | 1 |
| Median (min/max) | 3.3 (3.3–3.3) | 20.0 (10.0–1333.3) | 100.0 (100.0–100.0) |
| Eliciting dose |  |  |  |
| Screening DBPCFC, n | 3 | 3 | 1 |
| Median, mg (range) | 100.0 (10.0–100.0) | 100.0 (10.0–100.0) | 30.0 (30.0–30.0) |
| Exit DBPCFC, n | 2 | 3 | 1 |
| Median, mg (range) | 165.0 (30.0–300.0) | 1000.0 (600.0–4000.0) | 2000.0 (2000.0–2000.0) |
| *P*-value (Exit versus Screening DBPCFC)* | 0.500 | 0.250 | >0.999 |
| Fold change from Screening DBPCFC, n | 2 | 3 | 1 |
| Median (min/max) | 3.0 (3.0–3.0) | 10.0 (6.0–400.0) | 66.7 (66.7–66.7) |
| *P*-value (ADP101 versus placebo)† |  | 0.200 | 0.333 |
| **Hazelnut** |  |  |  |
| Participants with QFA, n | 3 | 0 | 3 |
| MTD |  |  |  |
| Screening DBPCFC, n | 3 | 0 | 3 |
| Median, mg (range) | 1.0 (1.0–3.0) |  | 10.0 (1.0–30.0) |
| Exit DBPCFC, n | 2 | 0 | 3 |
| Median, mg (range) | 2050.0 (100.0–4000.0) |  | 300.0 (300.0-4000.0) |
| Fold change from Screening DBPCFC, n | 2 | 0 | 3 |
| Median (min/max) | 716.7 (100.0–1333.3) |  | 300.0 (10.0–400.0) |
| Eliciting dose |  |  |  |
| Screening DBPCFC, n | 3 | 0 | 3 |
| Median, mg (range) | 1.0 (1.0–10.0) |  | 30.0 (1.0–100.0) |
| Exit DBPCFC, n | 2 | 0 | 3 |
| Median, mg (range) | 2150.0 (300.0–4000.0) |  | 600.0 (600.0–4000.0) |
| *P*-value (Exit versus Screening DBPCFC)* | 0.500 | NA | 0.250 |
| Fold change from Screening DBPCFC, n | 2 | 0 | 3 |
| Median (min/max) | 350.0 (300.0–400.0) |  | 133.3 (6.0–600.0) |
| *P*-value (ADP101 versus placebo)† |  | NA | 0.800 |
| **Peanut** |  |  |  |
| Participants with QFA, n | 11 | 9 | 12 |
| MTD |  |  |  |
| Screening DBPCFC, n | 11 | 9 | 12 |
| Median, mg (range) | 10.0 (3.0–30.0) | 10.0 (1.0–30.0) | 6.5 (1.0–30.0) |
| Exit DBPCFC, n | 10 | 6 | 10 |
| Median, mg (range) | 65.0 (3.0–2000.0) | 100.0 (3.0–4000.0) | 450.0 (100.0-4000.0) |
| Fold change from Screening DBPCFC, n | 10 | 6 | 10 |
| Median (min/max) | 3.2 (0.3–66.7) | 6.7 (1.0–400.0) | 66.7 (3.3–4000.0) |
| Eliciting dose |  |  |  |
| Screening DBPCFC, n | 11 | 9 | 12 |
| Median, mg (range) | 30.0 (10.0–100.0) | 30.0 (3.0–100.0) | 20.0 (3.0–100.0) |
| Exit DBPCFC, n | 10 | 6 | 10 |
| Median, mg (range) | 200.0 (10.0–4000.0) | 300.0 (10.0–4000.0) | 800.0 (300.0–4000.0) |
| *P*-value (Exit versus Screening DBPCFC)* | 0.047 | 0.063 | 0.002 |
| Fold change from Screening DBPCFC, n | 10 | 6 | 10 |
| Median (min/max) | 3.2 (0.3–40.0) | 6.5 (1.0–133.3) | 35.0 (3.0–1333.3) |
| *P*-value (ADP101 versus placebo)† |  | 0.366 | 0.004 |
| **Pecan** |  |  |  |
| Participants with QFA, n | 3 | 3 | 2 |
| MTD |  |  |  |
| Screening DBPCFC, n | 3 | 3 | 2 |
| Median, mg (range) | 3.0 (3.0–30.0) | 3.0 (1.0–3.0) | 1.0 (1.0–1.0) |
| Exit DBPCFC, n | 2 | 2 | 2 |
| Median, mg (range) | 55.0 (10.0–100.0) | 1050.0 (100.0–2000.0) | 100.0 (100.0–100.0) |
| Fold change from Screening DBPCFC, n | 2 | 2 | 2 |
| Median (min/max) | 16.8 (0.3–33.3) | 350.0 (33.3–666.7) | 100.0 (100.0–100.0) |
| Eliciting dose |  |  |  |
| Screening DBPCFC, n | 3 | 3 | 2 |
| Median, mg (range) | 10.0 (10.0–100.0) | 10.0 (3.0–10.0) | 2.0 (1.0–3.0) |
| Exit DBPCFC, n | 2 | 2 | 2 |
| Median, mg (range) | 165.0 (30.0–300.0) | 2150.0 (300.0–4000.0) | 300.0 (300.0–300.0) |
| *P*-value (Exit versus Screening DBPCFC)* | >0.999 | 0.500 | 0.500 |
| Fold change from Screening DBPCFC, n | 2 | 2 | 2 |
| Median (min/max) | 15.2 (0.3–30.0) | 215.0 (30.0–400.0) | 200.0 (100.0–300.0) |
| *P*-value (ADP101 versus placebo)† |  | 0.667 | 0.333 |
| **Pistachio** |  |  |  |
| Participants with QFA, n | 8 | 4 | 4 |
| MTD |  |  |  |
| Screening DBPCFC, n | 8 | 4 | 4 |
| Median, mg (range) | 10.0 (3.0–30.0) | 10.0 (10.0–30.0) | 10.0 (3.0–30.0) |
| Exit DBPCFC, n | 7 | 2 | 4 |
| Median, mg (range) | 100.0 (1.0–600.0) | 2300.0 (600.0–4000.0) | 2500.0 (600.0-4000.0) |
| Fold change from Screening DBPCFC, n | 7 | 2 | 4 |
| Median (min/max) | 3.3 (0.1–200.0) | 230.0 (60.0–400.0) | 250.0 (20.0–1333.3) |
| Eliciting dose |  |  |  |
| Screening DBPCFC, n | 8 | 4 | 4 |
| Median, mg (range) | 30.0 (10.0–100.0) | 30.0 (30.0–100.0) | 30.0 (10.0–100.0) |
| Exit DBPCFC, n | 7 | 2 | 4 |
| Median, mg (range) | 300.0 (1.0–1000.0) | 2500.0 (1000.0–4000.0) | 3000.0 (1000.0–4000.0) |
| *P*-value (Exit versus Screening DBPCFC)* | 0.156 | 0.500 | 0.125 |
| Fold change from Screening DBPCFC, n | 7 | 2 | 4 |
| Median (min/max) | 3.0 (0.1–100.0) | 83.3 (33.3–133.3) | 100.0 (10.0–400.0) |
| *P*-value (ADP101 versus placebo)† |  | 0.083 | 0.042 |
| **Salmon** |  |  |  |
| Participants with QFA, n | 0 | 1 | 0 |
| MTD |  |  |  |
| Screening DBPCFC, n | 0 | 1 | 0 |
| Median, mg (range) |  | 1.0 (1.0–1.0) |  |
| Exit DBPCFC, n | 0 | 1 | 0 |
| Median, mg (range) |  | 600.0 (600.0–600.0) |  |
| Fold change from Screening DBPCFC, n | 0 | 1 | 0 |
| Median (min/max) |  | 600.0 (600.0–600.0) |  |
| Eliciting dose |  |  |  |
| Screening DBPCFC, n | 0 | 1 | 0 |
| Median, mg (range) |  | 3.0 (3.0–3.0) |  |
| Exit DBPCFC, n | 0 | 1 | 0 |
| Median, mg (range) |  | 1000.0 (1000.0–1000.0) |  |
| *P*-value (Exit versus Screening DBPCFC)* | NA | >0.999 | NA |
| Fold change from Screening DBPCFC, n | 0 | 1 | 0 |
| Median (min/max) |  | 333.3 (333.3–333.3) |  |
| *P*-value (ADP101 versus placebo)† |  | NA | NA |
| **Sesame seed** |  |  |  |
| Participants with QFA, n | 0 | 2 | 1 |
| MTD |  |  |  |
| Screening DBPCFC, n | 0 | 2 | 1 |
| Median, mg (range) |  | 15.5 (1.0–30.0) | 1.0 (1.0–1.0) |
| Exit DBPCFC, n | 0 | 1 | 1 |
| Median, mg (range) |  | 3.0 (3.0–3.0) | 10.0 (10.0–10.0) |
| Fold change from Screening DBPCFC, n | 0 | 1 | 1 |
| Median (min/max) |  | 3.0 (3.0–3.0) | 10.0 (10.0–10.0) |
| Eliciting dose |  |  |  |
| Screening DBPCFC, n | 0 | 2 | 1 |
| Median, mg (range) |  | 51.5 (3.0–100.0) | 1.0 (1.0–1.0) |
| Exit DBPCFC, n | 0 | 1 | 1 |
| Median, mg (range) |  | 10.0 (10.0–10.0) | 30.0 (30.0–30.0) |
| *P*-value (Exit versus Screening DBPCFC)* | NA | >0.999 | >0.999 |
| Fold change from Screening DBPCFC, n | 0 | 1 | 1 |
| Median (min/max) |  | 3.3 (3.3–3.3) | 30.0 (30.0–30.0) |
| *P*-value (ADP101 versus placebo)† |  | NA | NA |
| **Shrimp** |  |  |  |
| Participants with QFA, n | 0 | 0 | 2 |
| MTD |  |  |  |
| Screening DBPCFC, n | 0 | 0 | 2 |
| Median, mg (range) |  |  | 6.5 (3.0–10.0) |
| Exit DBPCFC, n | 0 | 0 | 1 |
| Median, mg (range) |  |  | 100.0 (100.0–100.0) |
| Fold change from Screening DBPCFC, n | 0 | 0 | 1 |
| Median (min/max) |  |  | 10.0 (10.0–10.0) |
| Eliciting dose |  |  |  |
| Screening DBPCFC, n | 0 | 0 | 2 |
| Median, mg (range) |  |  | 20.0 (10.0–30.0) |
| Exit DBPCFC, n | 0 | 0 | 1 |
| Median, mg (range) |  |  | 300.0 (300.0–300.0) |
| *P*-value (Exit versus Screening DBPCFC)* | NA | NA | >0.999 |
| Fold change from Screening DBPCFC, n | 0 | 0 | 1 |
| Median (min/max) |  |  | 10.0 (10.0–10.0) |
| *P*-value (ADP101 versus placebo)† |  | NA | NA |
| **Soy** |  |  |  |
| Participants with QFA, n | 0 | 0 | 0 |
| **Walnut** |  |  |  |
| Participants with QFA, n | 5 | 3 | 3 |
| MTD |  |  |  |
| Screening DBPCFC, n | 5 | 3 | 3 |
| Median, mg (range) | 3.0 (1.0–30.0) | 3.0 (3.0–30.0) | 10.0 (3.0–30.0) |
| Exit DBPCFC, n | 3 | 2 | 2 |
| Median, mg (range) | 300.0 (1.0–300.0) | 50.5 (1.0–100.0) | 2150.0 (300.0–4000.0) |
| Fold change from Screening DBPCFC, n | 3 | 2 | 2 |
| Median (min/max) | 100.0 (0.1–100.0) | 1.8 (0.3–3.3) | 681.7 (30.0–1333.3) |
| Eliciting dose |  |  |  |
| Screening DBPCFC, n | 5 | 3 | 3 |
| Median, mg (range) | 10.0 (1.0–100.0) | 10.0 (10.0–100.0) | 30.0 (10.0–100.0) |
| Exit DBPCFC, n | 3 | 2 | 2 |
| Median, mg (range) | 600.0 (1.0–600.0) | 150.5 (1.0–300.0) | 2300.0 (600.0–4000.0) |
| *P*-value (Exit versus Screening DBPCFC)* | 0.500 | >0.999 | 0.500 |
| Fold change from Screening DBPCFC, n | 3 | 2 | 2 |
| Median (min/max) | 60.0 (0.0–60.0) | 1.6 (0.1–3.0) | 210.0 (20.0–400.0) |
| *P*-value (ADP101 versus placebo)† |  | 0.600 | 0.700 |
| **Wheat** |  |  |  |
| Participants with QFA, n | 0 | 0 | 2 |
| MTD |  |  |  |
| Screening DBPCFC, n | 0 | 0 | 2 |
| Median, mg (range) |  |  | 16.5 (3.0–30.0) |
| Exit DBPCFC, n | 0 | 0 | 2 |
| Median, mg (range) |  |  | 1015.0 (30.0–2000.0) |
| Fold change from Screening DBPCFC, n | 0 | 0 | 2 |
| Median (min/max) |  |  | 38.3 (10.0–66.7) |
| Eliciting dose |  |  |  |
| Screening DBPCFC, n | 0 | 0 | 2 |
| Median, mg (range) |  |  | 55.0 (10.0–100.0) |
| Exit DBPCFC, n | 0 | 0 | 2 |
| Median, mg (range) |  |  | 2050.0 (100.0–4000.0) |
| *P*-value (Exit versus Screening DBPCFC)* | NA | NA | 0.500 |
| Fold change from Screening DBPCFC, n | 0 | 0 | 2 |
| Median (min/max) |  |  | 25.0 (10.0–40.0) |
| *P*-value (ADP101 versus placebo)† |  | NA | NA |

Exploratory endpoint. *Unadjusted (nominal) *P*-value computed using Wilcoxon signed rank test. †Unadjusted (nominal) *P*-value computed using Wilcoxon rank sum test.
*DBPCFC, double-blind, placebo-controlled food challenge; ED, eliciting dose; max, maximum; min, minimum; MTD, maximum tolerated dose; QFA, qualifying food allergy; N, number of participants in the specified analysis population under each treatment group; n, number of participants in the specified group.*

## Table E8. Maximum severity of allergy symptoms at Screening and Exit DBPCFCs for qualifying foods (pediatric ITT population)

|  | **Screening DBPCFC** | | | **Exit DBPCFC** | | |
| --- | --- | --- | --- | --- | --- | --- |
| **Qualifying Food** | **Pooled placebo (N=20)**  **n (%)** | **LD ADP101**  **(N=21)**  **n (%)** | **HD ADP101 (N=20)**  **n (%)** | **Pooled placebo (N=20)**  **n (%)** | **LD ADP101**  **(N=21)**  **n (%)** | **HD ADP101 (N=20)**  **n (%)** |
| **Almond, N1** | **1** | **0** | **1** | **1** | **0** | **1** |
| Mild | 0 | 0 | 1 (100.0) | 0 | 0 | 1 (100.0) |
| Moderate | 1 (100.0) | 0 | 0 | 1 (100.0) | 0 | 0 |
| **Cashew, N1** | **10** | **7** | **5** | **9** | **5** | **4** |
| None | 0 | 0 | 0 | 1 (11.1) | 0 | 2 (50.0) |
| Mild | 4 (40.0) | 3 (42.9) | 1 (20.0) | 0 | 1 (20.0) | 1 (25.0) |
| Moderate | 6 (60.0) | 4 (57.1) | 3 (60.0) | 8 (88.9) | 4 (80.0) | 1 (25.0) |
| Severe | 0 | 0 | 1 (20.0) | 0 | 0 | 0 |
| **Chicken’s egg, N1** | **1** | **5** | **3** | **1** | **4** | **3** |
| None | 0 | 0 | 0 | 0 | 1 (25.0) | 0 |
| Moderate | 1 (100.0) | 5 (100.0) | 3 (100.0) | 1 (100.0) | 3 (75.0) | 2 (66.7) |
| Severe | 0 | 0 | 0 | 0 | 0 | 1 (33.3) |
| **Codfish, N1** | **0** | **0** | **2** | **0** | **0** | **2** |
| None | 0 | 0 | 0 | 0 | 0 | 1 (50.0) |
| Mild | 0 | 0 | 1 (50.0) | 0 | 0 | 0 |
| Moderate | 0 | 0 | 1 (50.0) | 0 | 0 | 1 (50.0) |
| **Cow’s milk, N1** | **3** | **3** | **1** | **2** | **3** | **1** |
| None | 0 | 0 | 0 | 0 | 1 (33.3) | 0 |
| Mild | 0 | 2 (66.7) | 1 (100.0) | 1 (50.0) | 0 | 0 |
| Moderate | 3 (100.0) | 1 (33.3) | 0 | 0 | 2 (66.7) | 1 (100.0) |
| Severe | 0 | 0 | 0 | 1 (50.0) | 0 | 0 |
| **Hazelnut, N1** | **3** | **0** | **3** | **2** | **0** | **3** |
| None | 0 | 0 | 0 | 1 (50.0) | 0 | 1 (33.3) |
| Mild | 1 (33.3) | 0 | 2 (66.7) | 1 (50.0) | 0 | 0 |
| Moderate | 2 (66.7) | 0 | 1 (33.3) | 0 | 0 | 2 (66.7) |
| **Peanut, N1** | **11** | **9** | **12** | **10** | **6** | **10** |
| None | 0 | 0 | 0 | 0 | 1 (16.7) | 3 (30.0) |
| Mild | 3 (27.3) | 2 (22.2) | 5 (41.7) | 3 (30.0) | 2 (33.3) | 4 (40.0) |
| Moderate | 8 (72.7) | 7 (77.8) | 7 (58.3) | 7 (70.0) | 3 (50.0) | 3 (30.0) |
| **Pecan, N1** | **3** | **3** | **2** | **2** | **2** | **2** |
| Mild | 1 (33.3) | 2 (66.7) | 1 (50.0) | 0 | 1 (50.0) | 1 (50.0) |
| Moderate | 2 (66.7) | 1 (33.3) | 1 (50.0) | 2 (100.0) | 1 (50.0) | 1 (50.0) |
| **Pistachio, N1** | **8** | **4** | **4** | **7** | **2** | **4** |
| None | 0 | 0 | 0 | 0 | 1 (50.0) | 2 (50.0) |
| Mild | 3 (37.5) | 3 (75.0) | 1 (25.0) | 0 | 0 | 1 (25.0) |
| Moderate | 5 (62.5) | 1 (25.0) | 3 (75.0) | 7 (100.0) | 1 (50.0) | 1 (25.0) |
| **Salmon, N1** | **0** | **1** | **0** | **0** | **1** | **0** |
| Moderate | 0 | 1 (100.0) | 0 | 0 | 1 (100.0) | 0 |
| **Sesame seed, N1** | **0** | **2** | **1** | **0** | **1** | **1** |
| Mild | 0 | 1 (50.0) | 0 | 0 | 1 (100.0) | 0 |
| Moderate | 0 | 0 | 1 (100.0) | 0 | 0 | 1 (100.0) |
| Severe | 0 | 1 (50.0) | 0 | 0 | 0 | 0 |
| **Shrimp, N1** | **0** | **0** | **2** | **0** | **0** | **1** |
| Moderate | 0 | 0 | 2 (100.0) | 0 | 0 | 1 (100.0) |
| **Soy, N1** | **0** | **0** | **0** | **0** | **0** | **0** |
| **Walnut, N1** | **5** | **3** | **3** | **3** | **2** | **2** |
| None | 0 | 0 | 0 | 0 | 0 | 1 (50.0) |
| Mild | 0 | 1 (33.3) | 2 (66.7) | 0 | 2 (100.0) | 0 |
| Moderate | 5 (100.0) | 2 (66.7) | 1 (33.3) | 3 (100.0) | 0 | 1 (50.0) |
| **Wheat, N1** | **0** | **0** | **2** | **0** | **0** | **2** |
| Moderate | 0 | 0 | 2 (100.0) | 0 | 0 | 2 (100.0) |

Exploratory endpoint. Severity rows containing all zeros not shown.
*DBPCFC, double-blind, placebo-controlled food trial; HD, high-dose; LD, low-dose; N, number of participants in the specified analysis population under each treatment group; N1, number of participants with the qualifying food allergy at Screening or Exit.*

## Table E9. Summary of accidental exposures (pediatric ITT population)

| **Category, n (%)** | **Pooled placebo (N=20)** | **LD ADP101**  **(N=21)** | **HD ADP101 (N=20)** |
| --- | --- | --- | --- |
| **Total number of accidental exposures** | 8 | 2 | 7 |
| Participants with any accidental exposures | 4 (20.0) | 2 (9.5) | 5 (25.0) |
| Participants with accidental exposures requiring treatment | 3 (15.0) | 0 | 4 (20.0) |
| Participants with accidental exposures requiring epinephrine use | 1 (5.0) | 0 | 1 (5.0) |
| Participants with accidental exposures requiring hospitalization | 0 | 0 | 0 |
| Participants with adverse events due to accidental exposure | 4 (20.0) | 1 (4.8) | 5 (25.0) |

Exploratory endpoint. *ITT, intent-to-treat; LD, low-dose; HD, high-dose; N, number of participants in the specified analysis population under each treatment group.*

## Table E10. Day 1 TEAEs, and TEAEs occurring in ≥2 participants in any treatment group by treatment phase (pediatric safety population)

|  | **Day 1 of up-dosing (single 5 mg dose of ADP101 or placebo)** | | | | | | | | |
| --- | --- | --- | --- | --- | --- | --- | --- | --- | --- |
| **TEAEs occurring in any treatment arm, n (%)** | **Pooled placebo (N=20)** | | **LD ADP101**  **(N=21)** | | | | **HD ADP101 (N=20)** | | |
| **Participants with ≥1 TEAE on day 1** | **2 (10)** | | **2 (10)** | | | | **2 (10)** | | |
| Back discomfort | 1 (5.0) | | 0 | | | | 0 | | |
| Emesis | 0 | | 1 (4.8) | | | | 0 | | |
| Influenza A virus infection | 0 | | 0 | | | | 1 (5.0) | | |
| Lip erythema | 0 | | 1 (4.8) | | | | 0 | | |
| Tingling throat | 0 | | 0 | | | | 1 (5.0) | | |
| Viral syndrome | 1 (5.0) | | 0 | | | | 0 | | |
|  | **Up-dosing phase** | | | | **Dose-maintenance phase** | | | | |
| **TEAEs occurring in ≥2 participants in any treatment arm, n (%)** | **Pooled placebo (N=20)** | **LD ADP101**  **(N=21)** | | **HD ADP101 (N=20)** | | **Pooled placebo (N=20)** | | **LD ADP101**  **(N=21)** | **HD ADP101 (N=20)** |
| **Participants with ≥1 TEAE** | **15 (75.0)** | **17 (81.0)** | | **17 (85.0)** | | **11 (55.0)** | | **12 (57.1)** | **13 (65.0)** |
| COVID-19 | 2 (10.0) | 4 (19.0) | | 6 (30.0) | | 3 (15.0) | | 1 (4.8) | 0 |
| Anaphylactic reaction | 3 (15.0) | 4 (19.0) | | 6 (30.0) | | 1 (5.0) | | 0 | 1 (5.0) |
| Vomiting | 2 (10.0) | 5 (23.8) | | 3 (15.0) | | 2 (10.0) | | 1 (4.8) | 2 (10.0) |
| Oral pruritus | 1 (5.0) | 4 (19.0) | | 4 (20.0) | | 1 (5.0) | | 1 (4.8) | 3 (15.0) |
| Urticaria | 4 (20.0) | 3 (14.3) | | 5 (25.0) | | 0 | | 1 (4.8) | 0 |
| Abdominal pain | 3 (15.0) | 5 (23.8) | | 2 (10.0) | | 1 (5.0) | | 2 (9.5) | 0 |
| Abdominal pain upper | 3 (15.0) | 3 (14.3) | | 3 (15.0) | | 2 (10.0) | | 0 | 2 (10.0) |
| Cough | 3 (15.0) | 3 (14.3) | | 2 (10.0) | | 1 (5.0) | | 1 (4.8) | 3 (15.0) |
| Upper respiratory tract infection | 1 (5.0) | 3 (14.3) | | 3 (15.0) | | 2 (10.0) | | 2 (9.5) | 2 (10.0) |
| Pruritus | 2 (10.0) | 5 (23.8) | | 4 (20.0) | | 0 | | 1 (4.8) | 0 |
| Nasal congestion | 2 (10.0) | 2 (9.5) | | 3 (15.0) | | 0 | | 1 (4.8) | 3 (15.0) |
| Throat irritation | 1 (5.0) | 4 (19.0) | | 3 (15.0) | | 1 (5.0) | | 1 (4.8) | 0 |
| Diarrhea | 5 (25.0) | 2 (9.5) | | 0 | | 2 (10.0) | | 1 (4.8) | 0 |
| Nausea | 1 (5.0) | 5 (23.8) | | 1 (5.0) | | 0 | | 1 (4.8) | 2 (10.0) |
| Abdominal discomfort | 0 | 4 (19.0) | | 2 (10.0) | | 0 | | 2 (9.5) | 2 (10.0) |
| Headache | 3 (15.0) | 2 (9.5) | | 1 (5.0) | | 0 | | 2 (9.5) | 0 |
| Viral infection | 3 (15.0) | 1 (4.8) | | 2 (10.0) | | 0 | | 1 (4.8) | 1 (5.0) |
| Oral paresthesia | 0 | 5 (23.8) | | 0 | | 1 (5.0) | | 1 (4.8) | 0 |
| Nasopharyngitis | 2 (10.0) | 2 (9.5) | | 0 | | 3 (15.0) | | 0 | 0 |
| Viral gastroenteritis | 1 (5.0) | 2 (9.5) | | 1 (5.0) | | 1 (5.0) | | 1 (4.8) | 0 |
| Erythema | 1 (5.0) | 3 (14.3) | | 1 (5.0) | | 0 | | 0 | 0 |
| Oropharyngeal pain | 2 (10.0) | 1 (4.8) | | 0 | | 0 | | 1 (4.8) | 1 (5.0) |
| Pyrexia | 1 (5.0) | 1 (4.8) | | 0 | | 2 (10.0) | | 1 (4.8) | 0 |
| Dyspnea | 0 | 1 (4.8) | | 2 (10.0) | | 0 | | 1 (4.8) | 0 |
| Throat tightness | 1 (5.0) | 0 | | 2 (10.0) | | 0 | | 0 | 1 (5.0) |
| Pharyngeal paresthesia | 0 | 2 (9.5) | | 1 (5.0) | | 0 | | 0 | 0 |
| Rhinorrhea | 0 | 1 (4.8) | | 2 (10.0) | | 0 | | 0 | 0 |
| Tongue pruritis | 0 | 2 (9.5) | | 1 (5.0) | | 0 | | 0 | 0 |
| Chest discomfort | 0 | 2 (9.5) | | 0 | | 0 | | 0 | 1 (5.0) |
| Lip swelling | 0 | 2 (9.5) | | 0 | | 0 | | 1 (4.8) | 0 |
| Arthropod sting | 0 | 0 | | 0 | | 2 (10.0) | | 0 | 1 (5.0) |
| Seasonal allergy | 2 (10.0) | 0 | | 0 | | 0 | | 0 | 0 |

Allergic TEAEs graded based on the CoFAR Grading Scale v1; nonallergic TEAEs graded based on the National Cancer Institute CTCAE version 5.0.
*CoFAR, Consortium of Food Allergy Research; CTCAE, Common Terminology Criteria for Adverse Events; HD, high-dose; LD, low-dose; N, number of participants in the specified analysis population under each group; n, number of participants in the specified group; TEAE, treatment-emergent adverse events.*

## **Table E11. TEAEs reported by ≥20% of participants in any treatment group (pediatric population)**

| **System organ class/preferred term n (%)** | **Pooled placebo (N=20)** | **LD ADP101**  **(N=21)** | **HD ADP101 (N=20)** |
| --- | --- | --- | --- |
| **Participants with ≥1 TEAE** | **19 (95.0)** | **20 (95.2)** | **19 (95.0)** |
| **Gastrointestinal disorders** | **14 (70.0)** | **19 (90.5)** | **15 (75.0)** |
| Abdominal pain | 6 (30.0) | 10 (47.6) | 6 (30.0) |
| Nausea | 2 (10.0) | 9 (42.9) | 7 (35.0) |
| Oral pruritus | 5 (25.0) | 5 (23.8) | 7 (35.0) |
| Abdominal pain upper | 4 (20.0) | 4 (19.0) | 7 (35.0) |
| Vomiting | 4 (20.0) | 6 (28.6) | 5 (25.0) |
| Diarrhoea | 7 (35.0) | 5 (23.8) | 1 (5.0) |
| Oral paresthesia | 2 (10.0) | 8 (38.1) | 2 (10.0) |
| Abdominal discomfort | 1 (5.0) | 5 (23.8) | 4 (20.0) |
| **Respiratory, thoracic and mediastinal disorders** | **11 (55.0)** | **14 (66.7)** | **13 (65.0)** |
| Nasal congestion | 2 (10.0) | 7 (33.3) | 7 (35.0) |
| Cough | 5 (25.0) | 6 (28.6) | 3 (15.0) |
| Throat irritation | 5 (25.0) | 5 (23.8) | 4 (20.0) |
| **Infections and infestations** | **12 (60.0)** | **11 (52.4)** | **14 (70.0)** |
| COVID-19 | 6 (30.0) | 6 (28.6) | 7 (35.0) |
| Upper respiratory tract infection | 2 (10.0) | 4 (19.0) | 5 (25.0) |
| Nasopharyngitis | 4 (20.0) | 2 (9.5) | 1 (5.0) |
| **Skin and subcutaneous tissue disorders** | **11 (55.0)** | **12 (57.1)** | **10 (50.0)** |
| Urticaria | 7 (35.0) | 7 (33.3) | 6 (30.0) |
| Pruritus | 3 (15.0) | 7 (33.3) | 5 (25.0) |
| Erythema | 4 (20.0) | 3 (14.3) | 2 (10.0) |
| **Immune system disorders** | **12 (60.0)** | **8 (38.1)** | **10 (50.0)** |
| Anaphylactic reaction | 11 (55.0) | 8 (38.1) | 9 (45.0) |
| **Nervous system disorders** | **5 (25.0)** | **5 (23.8)** | **3 (15.0)** |
| Headache | 3 (15.0) | 5 (23.8) | 1 (5.0) |

System organ class/preferred term based on MedDRA v24.1.
*HD, high-dose; LD, low-dose;* *MedDRA, Medical Dictionary for Regulatory Activities; N, number of participants in the specified analysis population under each group; n, number of participants in the specified group; TEAE, treatment-emergent adverse event.*

## Table E12. TEAEs related to study drug in ≥2 participants in any treatment group, by treatment period (pediatric population)

|  | **Up-dosing phase** | | | **Dose-maintenance phase** | | |
| --- | --- | --- | --- | --- | --- | --- |
| **TEAEs occurring in ≥2 participants in any treatment arm, n (%)** | **Pooled placebo (N=20)** | **LD ADP101**  **(N=21)** | **HD ADP101 (N=20)** | **Pooled placebo (N=20)** | **LD ADP101**  **(N=21)** | **HD ADP101 (N=20)** |
| **Participants with ≥1 TEAE** | **11 (55.0)** | **16 (76.2)** | **12 (60.0)** | **2 (10.0)** | **4 (19.0)** | **6 (30.0)** |
| Oral pruritus | 1 (5.0) | 4 (19.0) | 4 (20.0) | 1 (5.0) | 1 (4.8) | 2 (10.0) |
| Anaphylaxis | 2 (10.0) | 3 (14.3) | 4 (20.0) | 0 | 0 | 0 |
| Grade 1–2 | 2 (10.0) | 3 (14.3) | 4 (20.0) | 0 | 0 | 0 |
| Grade 3 | 1 (5.0) | 0 | 0 | 0 | 0 | 0 |
| Grade 4–5 | 0 | 0 | 0 | 0 | 0 | 0 |
| Pruritus | 1 (5.0) | 5 (23.8) | 3 (15.0) | 0 | 1 (4.8) | 0 |
| Urticaria | 2 (10.0) | 2 (9.5) | 5 (25.0) | 0 | 1 (4.8) | 0 |
| Abdominal pain | 1 (5.0) | 5 (23.8) | 2 (10.0) | 1 (5.0) | 1 (4.8) | 0 |
| Abdominal pain upper | 2 (10.0) | 3 (14.3) | 2 (10.0) | 1 (5.0) | 0 | 2 (10.0) |
| Abdominal discomfort | 0 | 4 (19.0) | 2 (10.0) | 0 | 1 (4.8) | 2 (10.0) |
| Throat irritation | 1 (5.0) | 4 (19.0) | 2 (10.0) | 0 | 1 (4.8) | 0 |
| Vomiting | 1 (5.0) | 3 (14.3) | 3 (15.0) | 0 | 0 | 1 (5.0) |
| Nausea | 1 (5.0) | 4 (19.0) | 0 | 0 | 1 (4.8) | 2 (10.0) |
| Oral paresthesia | 0 | 5 (23.8) | 0 | 0 | 1 (4.8) | 0 |
| Erythema | 0 | 3 (14.3) | 1 (5.0) | 0 | 0 | 0 |
| Nasal congestion | 0 | 1 (4.8) | 2 (10.0) | 0 | 0 | 1 (5.0) |
| Pharyngeal paresthesia | 0 | 2 (9.5) | 1 (5.0) | 0 | 0 | 0 |
| Throat tightness | 1 (5.0) | 0 | 2 (10.0) | 0 | 0 | 0 |
| Tongue pruritis | 0 | 2 (9.5) | 1 (5.0) | 0 | 0 | 0 |
| Cough | 2 (10.0) | 0 | 0 | 0 | 0 | 1 (5.0) |
| Chest discomfort | 0 | 2 (9.5) | 0 | 0 | 0 | 1 (5.0) |

Allergic TEAEs were graded based on the CoFAR Grading Scale v1. *CoFAR, Consortium of Food Allergy Research; HD, high-dose; LD, low-dose; N, number of participants in the specified analysis population under each group; n, number of participants in the specified group; TEAE, treatment-emergent adverse event.*

## Table E13. Food equivalents in milligrams of protein

| **Food** | **Protein/**  **serving** | **1**  **mg** | **3**  **mg** | **10**  **mg** | **30**  **mg** | **100**  **mg** | **300**  **mg** | **600**  **mg** | **1000**  **mg** | **2000**  **mg** | **4000**  **mg** |
| --- | --- | --- | --- | --- | --- | --- | --- | --- | --- | --- | --- |
| **Almond** | 3 g / 11 whole nuts | 1/270 nut | 1/90 nut | 1/27 nut | 1/9 nut | 4/11 nut | 1.1 nut | 2.2 nuts | 3.7 nuts | 7.3 nuts | 14.7 nuts |
| **Cashew** | 3 g / 10 whole nuts | 1/300 nut | 1/100 nut | 1/30 nut | 1/10 nut | 1/3 nut | 1 nut | 2 nuts | 3.3 nuts | 6.7 nuts | 13.3 nuts |
| **Chicken’s**  **egg** | 6 g / 1  large egg | 1/6000  egg | 1/2000  egg | 1/600  egg | 1/200  egg | 1/60  egg | 1/20  egg | 1/10  egg | 1/6  egg | 1/3  egg | 2/3  egg |
| **Codfish** | 6 g / 1 oz cooked fish | 1/6000 oz fish | 1/2000 oz  fish | 1/600 oz  fish | 1/200 oz  fish | 1/60 oz  fish | 1/20 oz  fish | 1/10 oz  fish | 1/6 oz fish | 1/3 oz fish | 2/3 oz  fish |
| **Cow’s**  **milk** | 8 g / 8 oz milk or yogurt | 0.002 tbsp yogurt | 0.006 tbsp yogurt | 0.02 tbsp yogurt | 0.06 tbsp yogurt | 0.2 tbsp yogurt | 0.6 tbsp yogurt | 1.2 tbsp yogurt | 2 tbsp yogurt | 4 tbsp yogurt | 8 tbsp yogurt |
| **Hazelnut** | 3 g /  3 tbsp nuts | 1/1000 tbsp nuts | 3/1000 tbsp nuts | 1/100  tbsp nuts | 3/100  tbsp nuts | 1/10  tbsp nuts | 3/10  tbsp nuts | 6/10  tbsp nuts | 1  tbsp nuts | 2  tbsp nuts | 4  tbsp nuts |
| **Peanut** | 2 g /  ~8 peanuts | 1/250 peanut | 3/250 peanut | 1/25 peanut | 3/25  peanut | 2/5  peanut | 1.2 peanuts | 2.4 peanuts | 4  peanuts | 8  peanuts | 16  peanuts |
| **Pecan** | 3 g /  25 halves | 5/600 halve | 1/250 halve | 5/60  halve | 1/4 halve | 5/6  halve | 2.5  halves | 5  halves | 8  halves | 16.7  halves | 33.3  halves |
| **Pistachio** | 3 g / 20 whole nuts | 1/150  nut | 1/50  nut | 1/15  nut | 1/5  nut | 2/3  nut | 2  nuts | 4  nuts | 6.7  nuts | 13.3  nuts | 26.7  nuts |
| **Salmon** | 6 g / 1 oz cooked fish | 1/6000 oz fish | 1/2000 oz  fish | 1/600 oz  fish | 1/200 oz  fish | 1/60 oz  fish | 1/20 oz  fish | 1/10 oz  fish | 1/6 oz fish | 1/3 oz fish | 2/3 oz  fish |
| **Sesame**  **Seed** | 3 g / 1 tbsp tahini | 1/3000 tbsp | 1/1000 tbsp | 1/300  tbsp | 1/100  tbsp | 1/30  tbsp | 1/10  tbsp | 2/10  tbsp | 1/3  tbsp | 2/3  tbsp | 1.3  tbsp |
| **Shrimp** | 5 g / 1 oz | 3/5000 medium shrimp | 9/5000 medium shrimp | 3/500  medium shrimp | 9/500 medium shrimp | 3/50 medium shrimp | 9/50 medium shrimp | 9/25 medium shrimp | 3/5 medium shrimp | 1.2 medium shrimp | 2.4 medium shrimp |
| **Soy** | 8 g / 3 oz firm tofu | 3/8000 oz tofu | 9/8000 oz tofu | 3/800 oz tofu | 9/800 oz  tofu | 3/80 oz tofu | 9/80 oz tofu | 9/40 oz  tofu | 3/8 oz  tofu | 3/4 oz  tofu | 1.5 oz  tofu |
| **Walnut** | 3 g /  10 halves | 1/300 halve | 1/100 halve | 1/30  halve | 1/10  halve | 1/3  halve | 1  halve | 2  halves | 3.3  halves | 6.7  halves | 13.3  halves |
| **Wheat** | 2–4 g /  slice bread | 1/4000 slice | 3/4000  slice | 1/400  slice | 3/400  slice | 1/40  slice | 3/40  slice | 3/20  slice | 1/4  slice | 1/2  slice | 1  slice |

Amounts are approximate; actual amounts of protein will vary by specific food and preparation. Table does not reflect DBPCFC offerings used in Harmony. Protein per serving based on Bird et al (2020). ^2^ *DBPCFC, double-blind, placebo-controlled food challenge; oz, ounce; tbsp, tablespoon.*

# References

1. Dickinson DF. The normal ECG in childhood and adolescence. *Heart*. 2005;91(12):1626-30. doi:10.1136/hrt.2004.057307

2. Bird JA, Leonard S, Groetch M, et al. Conducting an oral food challenge: An update to the 2009 Adverse Reactions to Foods Committee work group report. *J Allergy Clin Immunol Pract*. 2020;8(1):75-90.e17. doi:10.1016/j.jaip.2019.09.029
